# Quantitative in vitro-to-in vivo extrapolation of human adrenergic and trace amine-associated receptor 1 potencies of pre-workout supplement ingredients using physiologically based kinetic modelling-based reverse dosimetry

**DOI:** 10.1007/s00204-025-03992-7

**Published:** 2025-04-03

**Authors:** Nicole E. T. Pinckaers, W. Matthijs Blankesteijn, Anastasiya Mircheva, Ans Punt, Antoon Opperhuizen, Frederik-Jan van Schooten, Misha Vrolijk

**Affiliations:** 1 Department of Pharmacology and Toxicology, Maastricht University, Maastricht, The Netherlands; 2 Institute of Nutrition and Translational Research in Metabolism (NUTRIM), Maastricht University, Maastricht, The Netherlands; 3 Cardiovascular Research Institute Maastricht (CARIM), Maastricht University, Maastricht, The Netherlands; 4 Ans Punt Computational Toxicology, Arnhem, The Netherlands; 5 Office for Risk Assessment and Research, Netherlands Food and Consumer Product Safety Authority (NVWA), Utrecht, The Netherlands

**Keywords:** QIVIVE, Phenethylamine analogues, Adrenergic receptors, Trace amine-associated receptor 1, PBK modelling

## Abstract

The present study predicts effective doses of a set of phenethylamine (PEA) analogues that are frequently present in pre-workout and weight-loss food supplements, to prioritize these compounds for further risk assessment. In vitro determined EC_50_ values of PEA analogues for multiple human adrenergic receptor (ADR) subtypes (ADRα_1A_, α_1B_, α_1D_, α_2A_, β_1_, β_2_) and trace-amine associated receptor 1 (TAAR1) were extrapolated to human ED_50_ values by using physiologically based kinetic (PBK) modelling-based reverse dosimetry combined with in silico and in vitro determined PBK model input parameters. The predicted ED_50_ values of the studied PEAs for activation of ADRα_1A/B/D_, ADRα_2A_, ADRβ_1_ and TAAR1 were within a range of 0.914–29.7 mg/kg body weight (bw), 139–234 mg/kg bw, 0.0839–38.8 mg/kg bw and 0.995–264 mg/kg bw, respectively. Comparison of the predicted ED_50_ values with reported intake values revealed that particularly the exposure of the PEA analogues higenamine, isopropyloctopamine, β-methylphenethylamine and p-synephrine is in the same range or exceeds the predicted ED_50_ values. This suggests that these PEAs can (in)directly affect the cardiovascular system after the intake of food supplements. These PEA analogues should therefore be considered as high priority compounds for further risk assessment. In conclusion, our study shows that the use of quantitative in vitro-to-in vivo extrapolation (QIVIVE) of adrenergic and TAAR1 potencies using a generic PBK model can serve as an efficient prioritization method for a whole set of chemical analogues.

**Supplementary Information:**

The online version contains supplementary material available at 10.1007/s00204-025-03992-7.

## Introduction

**Fig. 1:**
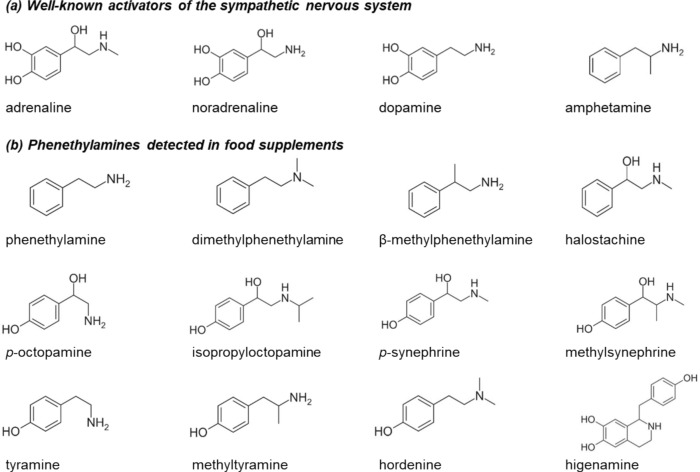
Molecular structures of (a) well-known activators of the sympathetic nervous system and (b) phenethylamine and its analogues detected in food supplements

The use of food supplements containing pharmacologically active substances to enhance sports performance and weight loss is increasing (Razenberg et al. 2021; Wardenaar et al. 2014). Among these substances, phenethylamine (PEA) analogues comprise a large group of compounds that are frequently used in such supplements (Avula et al. 2019; Biesterbos et al. 2019). PEA is a biogenic amine that is structurally related to the stimulant amphetamine and to the endogenous catecholamines (nor)adrenaline and dopamine (Fig. 1). The use of supplements containing PEA analogues has been associated with numerous adverse cardiovascular events, including palpitations, tachycardia, myocardial infarct and hemorrhagic stroke (Bovee et al. 2016; Cohen et al. 2015a; de Jonge et al. 2023; Harris et al. 2017; Smith et al. 2014). Surprisingly, there generally is a lack of knowledge regarding the toxicological mechanisms and effects of these substances upon exposure. Despite the scarcity of available information, supplements containing PEA analogues are widely available on the market, are still being used on a large scale and are even becoming more popular in the last few years (Razenberg et al. 2021; Wardenaar et al. 2014). This urges for better regulation of such supplements to protect consumers against their potential adverse health effects.

The detrimental effects of the PEA analogues on the cardiovascular system are suggested to be the result of mimicking activation of the sympathetic nervous system via various mechanisms (Broadley 2010); (Pinckaers et al. 2024; Rickli et al. 2019). Previous research showed that many PEA analogues are able to activate one or multiple adrenergic receptors (ADRs) and/or the trace amine-associated receptor 1 (TAAR1) in vitro (Pinckaers et al. 2024). These receptors play a critical role in cardiovascular homeostasis by regulating vascular smooth muscle- and cardiac contraction (Graham 1990). However, it is hard to causally link the adverse health effects to exposure to these compounds, as in vivo effects and kinetic data for most of these compounds are lacking. Consequently, it is not possible to perform a thorough risk assessment of these PEA analogues.

To stimulate proper regulation and risk communication of the use of these potentially hazardous substances in food supplements, there is a need for data on their safety profile. In the past, experimental animal studies would predominantly be performed to obtain toxicological data on chemical substances and used to predict their safety for humans. However, during the last decades, food and chemical risk assessment are more and more reducing the use of animal studies for ethical, economical and translatability reasons and replacing them with new techniques to assess chemical toxicity, also known as New Approach Methodologies (NAMs). NAMs include any in vitro, in silico or chemistry-based method that may be used to acquire information that could be valuable for chemical safety assessment for instance by predicting the point of departure for safe exposure levels in humans (Fischer et al. 2020).

An example of such a NAM is a physiologically based kinetic (PBK) model: an in silico tool that predicts absorption, distribution, metabolism and excretion (ADME) (Louisse et al. 2017). PBK modelling-based reverse dosimetry enables the translation of in vitro effect concentrations to in vivo effects doses. This quantitative in vitro-to-in vivo extrapolation (QIVIVE) can serve as an approach to prioritize compounds for additional risk assessment and in this way accelerate human health safety assessment (Paul Friedman et al. 2019). Currently, there is a need for case studies to gain confidence in PBK model predictions on the way towards regulatory acceptance of these models (Najjar et al. 2022; Otto et al. 2023). In the present study, a set of PEA analogues, that are frequently present in pre-workout and weight loss food supplements (Fig. 1b), is selected as a case for the application of the QIVIVE of adrenergic and TAAR1 potencies. In vitro determined EC_50_ values of PEA analogues for ADRα_1A_, α_1B_, α_1D_, α_2A_, β_1_, β_2_ and TAAR1 are extrapolated to ED_50_ values by using PBK modelling-based reverse dosimetry combined with in silico and in vitro determined PBK model input parameters.

## Materials and methods

### Chemicals

Β-methylphenethylamine (99%), phenethylamine (99.7%), tyramine hydrochloride (≥ 98%), halostachine (99%), hordenine (≥ 97.5%), octopamine (≥ 95%), uridine 5’-diphosphoglucuronic acid trisodium salt (UDPGA), alamethicin, adenosine 3’-phosphate 5’-phosphosulfate lithium salt hydrate (PAPS), S-(5’-Adenosyl)-L-methionine *p*-toluenesulfonate salt (SAM), HEPES and ammonium formate were purchased from Sigma-Aldrich (St. Louis, USA). Synephrine hydrochloride (99.6%) and higenamine hydrochloride (98.98%) were purchased from MedChemExpress (Monmouth Junction, USA). Methyltyramine (97%) was purchased from Apollo Scientific (Stockport, United Kingdom). Sodium bicarbonate, dipotassium phosphate, monopotassium phosphate, magnesium chloride hexahydrate and formic acid (98–100%) were purchased from Merck (Darmstadt, Germany). Methylsynephrine hydrochloride (99.8%) was purchased from Mikromol (Luckenwalde, Germany), isopropyloctopamine acetate (99.9%) from Honeywell Fluka (Seetze, Germany), isopropyloctopamine hydrochloride (98%) from Toronto Research Chemicals (Toronto, Canada), N,N-dimethylphenethylamine hydrochloride (> 99%) from GLPBio (Montclair, USA), metoprolol tartrate from EDQM (Strasbourg, France), acetonitrile from Biosolve (Valkenswaard, The Netherlands) and dimethylsulfoxide (DMSO) from Carl Roth (Karlsruhe, Germany). Nicotinamide adenine dinucleotide phosphate tetrasodium salt (NADPH) was purchased from Roche Diagnostics (Mannheim, Germany).

### Caco-2 permeability assays

To predict the human effective intestinal permeability (P_eff_) and the intestinal uptake rate constant (k_a_) of the phenethylamine analogues, Caco-2 permeability coefficients (P_app_) were determined. Caco-2 cells (ATCC, Manassas, VA, USA; passage 30–44) were cultured in DMEM (Gibco, New York, USA) containing 4.5 g/L d-glucose, 4 mM L-glutamine and supplemented with 10% FBS (Gibco, Grand Island, NY, USA), 1% minimal essential medium non-essential amino acids (Gibco, Paisley, UK), 100 units/mL penicillin and 100 µg/mL streptomycin (Gibco, Grand Island, NY, USA). Cells were seeded at a density of 4.0 × 10^4^ cells/cm^2^ on polyester transwell insert membranes (12 mm insert, 0.4 µm pore size; Corning incorporated, Kennebunk, ME, USA). Cells were maintained in a humidified incubator with 5% CO_2_ at 37 °C for 21 or 22 days in 0.5 mL and 1.5 mL medium in the apical and basolateral compartments, respectively. The medium was refreshed every two or three days and always one day before the permeability assay.

On the day of the experiment, cells were washed twice with HBSS without phenol red (pH 7.4; Sigma-Aldrich, Steinheim, Germany) supplemented with 0.35 g/L sodium bicarbonate and 25 mM HEPES and they were equilibrated in this supplemented HBSS for 15 min in a humidified incubator with 5% CO_2_ at 37 °C. Stocks of 10 mM PEAs and metoprolol, which was used as a reference compound, were prepared in DMSO. These stocks were diluted to a final concentration of 10 µM in pre-warmed supplemented HBSS (final DMSO level = 0.1%). After 15 min, the transepithelial electrical resistance (TEER) was measured with an Epithelial Voltohmmeter (World Precision Instruments, Sarasota, USA). Compound transport was studied in two directions: from the apical to the basolateral compartment (A-B) and from the basolateral to the apical compartment (B-A). For A-B experiments, 450 µL of the compound (10 µM) was added to the apical side and 1200 µL supplemented HBSS was added to the basolateral side. A sample of 50 µL was removed immediately from the apical compartment at time point zero. After 15 and 30 min, samples were taken from the basolateral compartment. Cells were kept in the incubator during the experiment. After 60 min, medium from both compartments was sampled, cells were washed twice with supplemented HBSS and were maintained in the incubator. After 15 min, the TEER was measured again to check for epithelial barrier damage due to compound exposure. After this, cells were lysed in RIPA buffer (Thermo Scientific, Rockford, IL, USA). For B-A experiments, 400 µL of supplemented HBSS was added to the apical side and 1250 µL of the compound (10 µM) was added to the basolateral side. A sample of 50 µL was removed immediately from the basolateral compartment at time point zero. After 15 and 30 min, samples were taken from the apical compartment. After 60 min, samples were taken similarly as in A-B experiments. The sampled medium and cell lysates were stored at −20 °C till the day of the liquid chromatography–mass spectrometry (LC–MS/MS) analysis. All experiments were run in duplicate. The compounds were quantified with LC–MS/MS as described in Sect. "LC–MS/MS analysis".

Before chemical analysis, cell lysates were two times diluted in ice-cold methanol and centrifuged at 15,000 rpm at 4 °C. The supernatant was then injected into the LC–MS/MS system. Sampled buffer from donor compartments was diluted 40 times in buffer and samples from the receiver compartment were analysed undiluted. Standards were prepared in the same assay buffer to quantify the amount of compound in medium samples. For quantification of compounds in cell lysates, standards were prepared in a mixture of 50% RIPA buffer and 50% methanol.

The effective intestinal permeability (P_app_) values were calculated according to the methods of Hubatsch et al. (2007):

$${\text{P}}_{{{\text{app}}}} \left( {{\text{cm}}/{\text{s}}} \right) = \left( {{{{\text{dQ}}} \mathord{\left/ {\vphantom {{{\text{dQ}}} {{\text{dt}}}}} \right. \kern-0pt} {{\text{dt}}}}} \right)({1 \mathord{\left/ {\vphantom {1 {\left( {{\text{A}}*{\text{C}}_{0} } \right)}}} \right. \kern-0pt} {\left( {{\text{A}}*{\text{C}}_{0} } \right)}}$$

where dQ/dt is the steady-state flux in µmol/s, A is the surface area of the cell culture insert membrane in cm^2^ and C_0_ is the starting concentration in µM in the donor compartment (apical compartment for A-B and basolateral compartment for B-A experiments). Efflux ratios were determined by P_app B-A_ / P_app A-B_. Caco-2 permeability assays are known to have high interlaboratory variability, which could be caused by Caco-2 passage number, filter membrane material, cell density and for example medium or assay buffer (Lee et al. 2017). To make our results comparable with other (future) studies, a correction factor for this interlaboratory variation was applied. All P_app A-B_ values used for the calculation of P_eff_ and k_a_ were corrected for the fold change of the P_app A-B_ of metoprolol in the current study and the average of the P_app A-B_ of metoprolol presented in the literature. As metoprolol is a widely studied compound, it is therefore chosen as a reference compound in the current study to correct for the aforementioned variables that may influence compound permeability. All data are presented as mean ± SD.

### In vitro hepatic clearance by human liver S9

In vitro intrinsic hepatic clearance (CL_int_) values of the phenethylamine analogues were determined in experiments with pooled human liver S9 (HLS9; Corning, Woburn, MA, USA and Gibco, USA), by measuring the rate of compound depletion. Stocks of 1 mM PEA compounds were prepared in DMSO and stocks of 10 mg/mL alamethicin were prepared in methanol. The final incubation mixtures consisted of 1 µM of PEA compound (0.1% DMSO) in 100 mM potassium phosphate buffer with 5 mM magnesium chloride (pH 7.4) supplemented with 0.025 mg/mL alamethicin (0.25% methanol) and 0.1–2 mg/mL HLS9. Optimal HLS9 concentrations for each compound were determined in separate pilot experiments by testing multiple HLS9 concentrations; the HLS9 concentration that showed a linear substrate depletion between 0 and 60 min was chosen. The optimal HLS9 concentrations are presented in Table 1. Incubation mixtures were first pre-incubated at 37 °C in a shaking incubator at 300 rpm for 5 min. After 5 min, the reaction was initiated by adding a co-factor mix in supplemented buffer with final concentrations of 3 mM nicotinamide adenine dinucleotide phosphate (NADPH; for oxidation, reduction and hydrolysis), 5 mM uridine diphosphate glucuronic acid (UDPGA; for glucuronidation), 0.2 mM adenosine 3’-phosphate 5’-phosphosulfate lithium salt hydrate (PAPS; for sulfation) and 0.2 mM S-(5’-Adenosyl)-L-methionine p-toluenesulfonate salt (SAM; for methylation). The final incubation volume was 100 µL and incubations were performed in 1.5 mL Safe-Lock Eppendorf tubes (Eppendorf) in a shaking incubator (300 rpm) at 37 °C (Eppendorf Thermomixer). Incubations were terminated by adding 100 µL ice-cold acetonitrile after, 0, 2.5, 5, 10, 20, 40 and 60 min. Then, samples were vortexed, put on ice and stored at -20 °C until the day of the LC–MS/MS analysis. Controls of incubation mixtures without HLS9 or mixtures without co-factors were also included. Two independent experiments, both in duplicate, were performed on different days. On the day of the LC–MS/MS analysis, samples were thawed and centrifuged at 15,000 rpm for 10 min. The supernatant was collected in glass insert vials for LC injection (BGB Analytik, Harderwijk, The Netherlands). Details on the methods of the LC–MS/MS analysis are described in Sect. "LC–MS/MS analysis". After determining the PEA (analogue) amount at the different time points, the CL_int_ was calculated. The natural logarithm of compound concentrations was plotted against time and the slope of this curve represented the elimination rate constant k (in 1/min). The half-life (t_1/2_ in minutes) was calculated by: ln (2)/k (in 1/min). The volume of incubation (V) in µL/mg protein was determined by: 1000 / HLS9 concentration (in mg/mL). Finally, CL_int_ could be calculated by:

$${\text{CL}}_{{{\text{int}}}} \left( {\mu {\text{L}}/{\text{min}}/{\text{mg protein}}} \right) = {\text{V}}\left( {\mu {\text{L}}/{\text{mg protein}}} \right)*\left( {{\text{ln }}\left( {2} \right)/{\text{t}}_{{{1}/{2}}} \left( {{\text{min}}} \right)} \right).$$

**Table 1:** Human liver S9 concentrations used in the incubations with the specific phenethylamine analogues

Compound	Human liver S9 concentration (mg/mL)
β-Methylphenethylamine	2
Dimethylphenethylamine	2
Halostachine	0.5
Higenamine	1
Hordenine	2
Isopropyloctopamine	2
Methylsynephrine	2
Methyltyramine	0.25
*p*-Octopamine	2
*p*-Synephrine	1
Phenethylamine	0.1
Tyramine	0.25

### In silico predicted input parameters

Physicochemical input data, such as pKa, logP and molecular weight (MW) were obtained from Chemicalize (April 2023, https://chemicalize.com/, developed by ChemAxon). Blood:plasma (B:P) ratios were predicted with the Simcyp ADME Prediction Toolbox V1.1 (Simcyp Limited 2012). The fraction unbound in plasma (F_up_) was predicted according to the method of (Lobell and Sivarajah 2003) and obtained via the QIVIVE toolbox from (Punt et al. 2021b) http://www.qivivetools.wur.nl). Tissue:plasma partition coefficients were simulated by the method of (Rodgers and Rowland 2006) and obtained via the QIVIVE toolbox (Punt et al. 2021b). The physicochemical properties of the PEAs that were used to predict tissue:plasma partition coefficients are presented in Table 2.

**Table 2:** Physicochemical properties of the phenethylamines used to calculate tissue:plasma partition coefficients

Compound	pKa^a^	logP	MW	Fraction unbound (F_up_)	Blood:plasma ratio
β-Methylphenethylamine	9.66	1.753	135.21	0.657	1.1638
Dimethylphenethylamine	9.41	2.203	149.237	0.541	1.1945
Halostachine	9.43	0.901	151.209	0.825	1.0416
Higenamine	8.57	2.35	271.316	0.484	1.2151
Hordenine	8.88	1.563	165.236	0.694	1.1442
Isopropyloctopamine	9.84	0.629	195.262	0.864	0.996
Methylsynephrine	9.8	0.284	181.235	0.902	0.9383
Methyltyramine	10.39	0.804	151.209	0.841	1.026
*p*-Octopamine	8.64	−0.131	153.181	0.93	0.8657
*p*-Synephrine	9.15	−0.071	167.208	0.929	0.8802
Phenethylamine	9.94	1.388	121.183	0.739	1.1187
Tyramine	9.34	0.605	137.182	0.866	0.991

^a^All compounds are monoprotic bases

The in vitro obtained Caco-2 P_app_ values were scaled to in vivo absorption rate constants (k_a_) and fraction absorbed (fa) with the following equations:

1 $${\text{log P}}_{{{\text{eff}}}} ,{\text{human}}\left( {{1}0^{{ - {4}}} {\text{cm}}/{\text{s}}} \right) = 0.{4926}*{\text{log P}}_{{{\text{app}}}} \left( {{1}0^{{ - {6}}} {\text{cm}}/{\text{s}}} \right){-}0.{1454}$$

2 $${\text{k}}_{{\text{a}}} \left( {/{\text{h}}} \right) = {\text{P}}_{{{\text{eff}}}} *{2}\left( {{\text{cm}}/{\text{s}}} \right)/{\text{R}}\left( {{\text{cm}}} \right)*{36}00\left( {{\text{s}}/{\text{h}}} \right)$$

3 $${\text{fa}} = {1}{-}({1} + \left( {{2}*{\text{P}}_{{{\text{eff}}}} * < {\text{Tsi}}> } \right)/\left( {{7}*{\text{R}}} \right)^{{ - {7}}}$$

in which P_eff_ is the effective permeability in humans, R is the intestinal radius and < Tsi > is the transit time in the small intestine. Equation (1) is based on the human study of (Sun et al. 2002), in which in vivo intestinal effective permeability is correlated to in vitro Caco-2 P_app_ values. Equations (2) and (3) are based on the methods of (Yu and Amidon 1999), in which R is 1 cm and is obtained from (Peters and Hultin 2008). For the < Tsi > a value of 3.32 h was used (Grandoni et al. 2019).

The fraction unbound in these in vitro liver clearance incubations was calculated with the method of (Hallifax and Houston 2006) in which unspecific binding in HLS9 incubations was assumed to be the same as in liver microsomal incubations. The in vitro obtained HLS9 CL_int_ values were corrected for protein binding in the HLS9 incubation and were scaled to a whole liver by using a scaling factor of 121 mg HLS9/g liver (Houston and Galetin 2008).

### PBK model code and software

The PBK model codes used in this study are presented in the Supplementary data. For the prediction of internal concentrations of the PEA analogues a generic PBK model for humans with oral dosing was used. The PBK model code was adopted from (Jones and Rowland-Yeo 2013). In general, the body compartments described in the model are: adipose, bone, brain, gut, heart, kidney, liver, lung, muscle, skin, spleen, testes, venous, arterial, plasma and a rest compartment. The concentrations in these compartments were modelled as blood-flow limited, in which it is assumed that the total concentration in the tissue is in equilibrium with the concentration in the systemic circulation at a steady state. The concentration in the circulation is compound-specific and is based on the plasma partition coefficients. The blood flow rate, the tissue volume and the plasma partition coefficients determine the rate of reaching the steady-state (Jones and Rowland-Yeo 2013). The model was converted to RStudio (version 2023.12.1 Build 402, “Ocean Storm Release”) solving the differential equations with the R rxode2 package (with tidyverse packages). C_liver free_ was defined as C_liver_/K_p liver_ * F_up_ instead of C_liver_* F_up_. In addition, the fraction absorbed (fa) accounted for the initial setting of the dose rather than the differential equation for oral absorption, as the latter would present the absorption rate and not the fraction that is absorbed. The renal clearance (urinary excretion) was simulated as glomerular filtration rate (GFR) times the free plasma concentration. This is an adjustment from the original model by Jones and Rowland-Yeo (2013), where renal clearance is described as CL_renal_*C_kidney free_ and it is not specified whether this renal clearance corresponds to urinary excretion or metabolic clearance. Furthermore, the plasma concentration was, in this study, converted to µmol/L or nmol/L as output of the model. The tissue partition coefficient of the testes and the remaining body compartment (K_p re_) was set equal to the muscle partition coefficient. Also, mass balance equations were added and V_rb_, V_plas_ven_ and V_plas_art_ were removed from the current model since these were not used in the current study.

The above-described generic PBK model was converted to a model with intravenous dosing. Intravenous administration was included in this study, as the in vivo available data of higenamine, which was used for the PBK model evaluation, was administered intravenously. The dosing regimen of the intravenous PBK model corresponds to the dosing regimen used in the study of (Feng et al. 2012), which was an escalating infusion of 0.5, 1, 2 and 4 µg/kg bw/min. The infusion duration of each dose was three minutes, resulting in a total dose of 22.5 µg/kg bw after 12 min of infusion.

### Sensitivity analysis

The influence of the PBK model input parameters on the modelled venous plasma concentration (C_plasma venous_) was investigated by performing a local sensitivity analysis. Normalised sensitivity coefficients (NSC) were calculated according to:

$${\text{NSC}} = \left( {{\text{C}}^{\prime } - {\text{C}}} \right)/\left( {{\text{P}} - {\text{P}}^{\prime } } \right)*\left( {{\text{P}}/{\text{C}}} \right)$$

in which C is the model output (i.e. C_plasma venous_) and P is the input parameter (Evans and Andersen 2000). P’ represents a 1% change of P and C’ represents the value of C when applying P’.

### Prediction of oral equivalent effect doses

The in vitro effect concentrations (EC_50_), for activation of the α_1A,_ α_1B,_ α_1D,_ α_2A,_ α_2B,_ β_1_ and β_2_ ADR activation and the activation of TAAR1, were obtained from our previous study (Pinckaers et al. 2024). These EC_50_ values were extrapolated to ED_50_ values in the current study as follow:

$${\text{predicted ED}}_{{{5}0}} = in \, vitro{\text{EC}}_{{{5}0}} *{\text{ 1 mg}}/{\text{kg bw }}/{\text{ modelled C}}_{{\text{max plasma venous}}} {\text{at 1 mg}}/{\text{kg bw}}_{.}$$

For this extrapolation, it was assumed that effects were only caused by the parent compound and not by its metabolites, and can be related to the peak plasma concentration.

### LC–MS/MS analysis

Samples from the Caco-2 permeability assays and the in vitro hepatic clearance assays were analysed with an ultra-high-performance liquid chromotograph (UPLC) (Agilent Technologies, Santa Clara, CA, USA) coupled to an Agilent 6550 iFunnel quadrupole-time-of-flight (Q-TOF) mass spectrometer equipped with a dual electrospray ionization (ESI) source in positive mode with Agilent Jet Stream technology. The injection volume was 5 µL for β-methylphenethylamine, isopropyloctopamine and metoprolol and was 2 µL for the remaining compounds. To measure β-methylphenethylamine, dimethylphenethylamine, phenethylamine, methyltyramine, higenamine, hordenine, methylsynephrine, isopropyloctopamine and metoprolol, the UPLC system was equipped with an Agilent Technologies InfinityLab Poroshell 120 EC-C18 column (2.1 × 100 mm, 2.7 µm pore size) with a similar guard column (2.1 × 5 mm, 2.7 µm pore size). Halostachine, *p*-octopamine, *p*-synephrine and tyramine were separated by an InfinityLab Poroshell 120 HILIC column (2.1 × 100 mm, 2.7 µm pore size) equipped with a similar guard column (2.1 × 5 mm, 2.7 µm pore size). The temperature of the C18 column wash held at 25 °C and of the HILIC column at 30 °C. The temperature of the autosampler was kept at 4 °C. For compounds that eluted from the C18 column, a gradient of 0.1% formic acid in water (A) and acetonitrile (B) was used. The gradient started at 5% B, was kept at 5% B for 1 min, and then linearly increased to 50% B in 10 min. After 2 min 50% B, the gradient linearly decreased to 5% B in 1 min and was kept at 5% B for another 4 min. For the compounds that eluted from the HILIC column, mobile phase A was 0.1% formic acid and 5 mM ammonium formate in water and mobile phase B was acetonitrile. The gradient started at 92% B, was kept at 92% B for 9 min, and then linearly decreased to 70% B in 0.5 min and kept at 70% B for 3 min. Then, the gradient linearly increased to 92% in 0.5 min and was kept at 92% B for 4 min.

A sheat gas temperature of 350 °C, a sheat gas flow of 660 L/h, a cone gas temperature of 200 °C and a cone gas flow of 840 L/h was used. The nebulizer pressure was set at 35 psi. The capillary voltage, the nozzle voltage and the fragmentor voltage were 3500, 1000 and 380, respectively. Nitrogen was used as a collision-induced dissociation gas. Collision energy was optimized for each compound. Retention time, collision energy and mass charge (m/z) transitions for each compound are described in Table 3. All ions were measured in positive ion mode (ESI +). Data acquisition was performed using Agilent MassHunter Workstation Quantitative Analysis for Q-TOF software version 10.2 (Agilent Technologies, Inc.).

**Table 3:** Compound-specific mass spectrometry parameters

Compound	Parent ion (m/z)	Daughter ion (m/z) (* = quantifier)	Collision energy (eV)	Retention time (minutes)
β-Methylphenethylamine	136.11	91.05 *	12.5	5.2
Dimethylphenethylamine	150.13	105.07 *	20	4.8
Halostachine	134.10 / 152.11	119.10 / 134.10 *	22.5 / 10	2.6
Higenamine	272.13	107.050 *	30	5.1
Hordenine	166.12	121.06 *	30	1.9
Isopropyloctopamine	178.12	91.05 *	30	2.3
Methylsynephrine	182.12 / 164.11	164.11 * / 149.08	10 / 20	1.4
Methyltyramine	152.1	121.06 *	8	1.8
Metoprolol	268.20	72.08 *	15	7.2
*p*-Octopamine	136.08	91.05 *	16	3.2
*p*-Synephrine	168.10 / 150.09	150.09 * / 135.07	22 / 5	3.2
Phenethylamine	122.09	105.07 *	8	3.7
Tyramine	121.06	77.0395 *	20	5.4

## Results

### Caco-2 permeability assays

Caco-2 permeability assays were performed to scale P_app_ values to k_a_ and fa, which were used as input values of the PBK model. Transport of the PEA analogues was studied in two directions: from the apical to the basolateral (A-B) compartment and vice versa (B-A). The efflux ratios were found within a range of 0.232 and 1.32 (Table 8 in appendix), indicating that no active efflux transporter was involved. P_app_ values were derived from cumulative amount-time profiles, which were linear for all compounds from 0 to 60 min, except for phenethylamine where it was linear from 0 to 30 min. (Fig. 7 in appendix). P_app A-B_ values for the PEA analogues were within a range of 0.0610 × 10^–6^ cm/s for higenamine to 15.8 × 10^–6^ cm/s for β-methylphenethylamine (Table 4). The recovery after 60 min for PEA in A-B experiments was 15.7 ± 1.51%. The recovery rates for the other PEA analogues in A-B experiments were within a range of 76.7 to 114% (Table 4). The mean P_app A-B_ of reference compound metoprolol in this study was 11.2 ± 0.197 × 10^–6^ cm/s (recovery = 103 ± 2.30%), whereas the mean P_app A-B_ of metoprolol reported in literature was 40.7 ± 35.5 × 10^–6^ cm/s (*n* = 20) with values ranging from 2.3 to 140 × 10^–6^ cm/s (Table 9 in appendix) (Alsenz and Haenel 2003; Artursson and Karlsson 1991; Galkin et al. 2008; Gertz et al. 2010; Hilgendorf et al. 2000; Incecayir et al. 2013; Irvine et al. 1999; Kerns et al. 2004; Lee et al. 2017; Lentz et al. 2000; Li et al. 2007; Neuhoff et al. 2003; Palm et al. 1996; Punt et al. 2021a; Skolnik et al. 2010; Teksin et al. 2010; Vaithianathan et al. 2015; Yang et al. 2007; Yazdanian et al. 1998; Zhu et al. 2002). The P_app A-B_ values of the PEAs were corrected for this interlaboratory difference before scaling them to k_a_ and fa (Table 4).

**Table 4:** Mean apparent permeability (*P*_*app A-B*_* ± SD*) values and recovery rates ± SD derived from the Caco-2 permeability assays; *P*_*app A-B*_ values are corrected for the difference between *P*_*app A-B*_ of reference compound metoprolol in this study and as reported in literature; the corrected P_app A-B_ values are scaled to absorption rate constants (k_a_) and fraction absorbed (fa) values

*Compound*	*P*_*app A-B*_ *(*× *10*^*–6*^* cm/s)*	*Recovery* *(%)*	*P*_*app A-B*_* corrected* *(*× *10*^*–6*^* cm/s)*	*k*_*a*_ *(/h)*	*fa*
β-Methylphenethylamine	15.8 ± 1.47	80.9 ± 3.27	57.6	3.79	1.00
Dimethylphenethylamine	14.7 ± 0.244	76.7 ± 0.06	53.5	3.66	1.00
Halostachine	13.1 ± 0.144	84.0 ± 3.18	47.8	3.46	1.00
Higenamine	0.0610 ± 0.0351	104 ± 0.35	0.223	0.246	0.860
Hordenine	13.0 ± 0.192	96.7 ± 1.27	47.3	3.46	1.00
Isopropyloctopamine	0.703 ± 0.0410	79.8 ± 2.19	2.56	0.818	0.994
Methylsynephrine	0.353 ± 0.215	114 ± 8.39	1.30	0.563	0.971
Methyltyramine	0.344 ± 0.0965	87.1 ± 9.14	1.26	0.576	0.981
*p*-Octopamine	0.164 ± 0.0701	98.9 ± 1.77	0.584	0.395	0.947
*p*-Synephrine	0.951 ± 0.497	109 ± 1.48	3.47	0.951	0.997
Phenethylamine	2.88 ± 0.220	15.7 ± 1.51	10.5	1.64	1.00
Tyramine	2.38 ± 1.186	114 ± 11.2	8.70	1.50	1.00

### In vitro hepatic clearance by human liver S9 (HLS9)

In vitro intrinsic hepatic clearance of the PEA analogues by HLS9 was determined in this study to scale to hepatic metabolic clearance (CL_met_), which was used as the input value of the PBK model. The compound depletion curves with the corresponding elimination rate constants (*k*) are presented in Fig. 8 in the appendix. The *k* values of incubation with HLS9 and co-factor mix were used to calculate the CL_int_ values, which are shown in Table 5. The apparent in vitro CL_int_ (CL_int app_) values range from 1.49 µL/min/mg protein for methylsynephrine to 241 µL/min/mg protein for PEA. These values are corrected for unspecific protein binding in the HLS9 incubation by using the correction method (Hallifax and Houston 2006). The predicted unbound fractions vary from 0.810 for methylsynephrine to 0.990 for phenethylamine, which results in corrected CL_int_ values (CL_int,u_) that did not differ more than 20% from the uncorrected values (Table 5). The CL_int, u_ was scaled to a whole liver, after which the hepatic metabolic clearance (CL_met_) was modelled (Table 5). The CL_met_ was the highest for phenethylamine (2598 L/h) and was the lowest for methylsynephrine (19.7 L/h). β-methylphenethylamine, dimethylphenethylamine, halostachine, methyltyramine, *p*-octopamine, *p*-synephrine, phenethylamine and tyramine were also metabolized in incubations without co-factor mix.

**Table 5:** In vitro *apparent intrinsic clearance (CL*_*int app*_*), predicted unbound HLS9 fractions and intrinsic clearance corrected for unspecific protein (HLS9) binding (CL*_*int, u*_*)*

Compound	In vitro CL_int app_ (µL/min/mg protein)	Predicted fraction unbound in incubation	In vitro CL_int,u_ (µL/min/mg protein)	Predicted intrinsic hepatic metabolic clearance CL_met_ (L/hr)
β-methylphenethylamine	12.1	0.930	13.0	139
Dimethylphenethylamine	20.0	0.870	23.0	245
Halostachine	37.4	0.960	39.0	416
Higenamine	22.0	0.900	24.4	261
Hordenine	2.20	0.870	2.53	27.0
Isopropyloctopamine	2.15	0.840	2.56	27.3
Methylsynephrine	1.49	0.810	1.84	19.7
Methyltyramine	118	0.970	122	1303
*p*-Octopamine	6.05	0.860	7.06	75.4
*p*-Synephrine	11.9	0.910	13.1	140
Phenethylamine	241	0.990	243	2598
Tyramine	77.2	0.980	78.8	841

### Sensitivity analysis

**Fig. 2:**
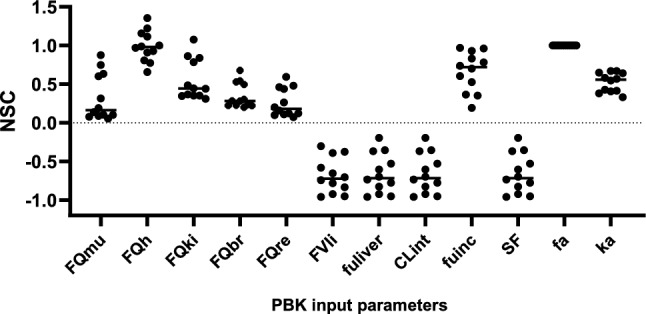
Normalised sensitivity coefficients (NSCs) of the C_max plasma venous_ simulations to the most critical input parameters for all studied phenethylamine (PEA) analogues at an oral dose of 1 mg/kg bw; for each parameter, data points of the 12 different PEA analogues are shown; FQmu, fractional muscle blood flow; FQh, fractional hepatic blood flow (venous side); FQki, fractional kidney blood flow; FQbr, fractional brain blood flow; FQre, fractional tissue blood flow rest of body; FVli, fractional liver volume; fuliver, fraction unbound in liver; Clint, in vitro intrinsic hepatic metabolic clearance; fuinc, fraction unbound in the in vitro liver S9 incubation; SF, scaling factor for mg liver S9 per gram liver; fa, fraction absorbed; ka, absorption rate constant

**Fig. 3:**
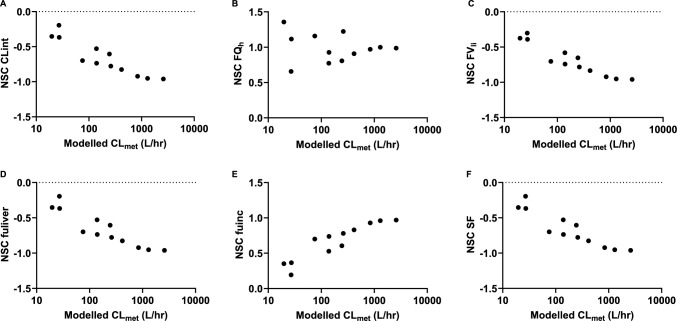
Correlations of Normalized Sensitivity Coefficients (NSCs) and the modelled intrinsic hepatic metabolic intrinsic clearance (CL_met_) for the NSCs of **(A)** CLint (= in vitro intrinsic hepatic metabolic clearance), **(B)** FQh (= fractional hepatic blood flow, venous side), **(C)** FVli (= fractional liver volume), **(D)** fuliver (= fraction unbound in liver), **(E)** fuinc (= fraction unbound in the in vitro liver S9 incubation) and **(F)** SF (= scaling factor for mg liver S9 per gram liver)

A local sensitivity analysis was performed to study the influence of the PBK model input parameters on the modelled maximal venous plasma concentrations (C_max plasma venous_) at an oral dose of 1 mg/kg bw. The NSCs obtained from this analysis for each compound are presented in Fig. 9 of the appendix. Figure 2 shows the NSCs of the most influential input parameters for all studied PEA analogues. Parameters that affected C_max plasma venous_ could be related to intestinal absorption (fa and ka) and hepatic metabolic clearance (CL_int_, fuinc, fuliver, FVli, SF and FQ_h_). The NSCs of most of the parameters related to hepatic metabolic clearance were increasing for compounds with increasing modelled clearance rates (CL_met_), with the exception of the FQh (Fig. 3). Other parameters that were critical included the fractional muscle, kidney, brain and rest tissue blood flow (FQmu, FQki, FQbr and FQre). The influence of input parameter fa was the same for all studied compounds (NSC = 1.00 ± 0.0000578).

### PBK model evaluation

**Fig. 4:**
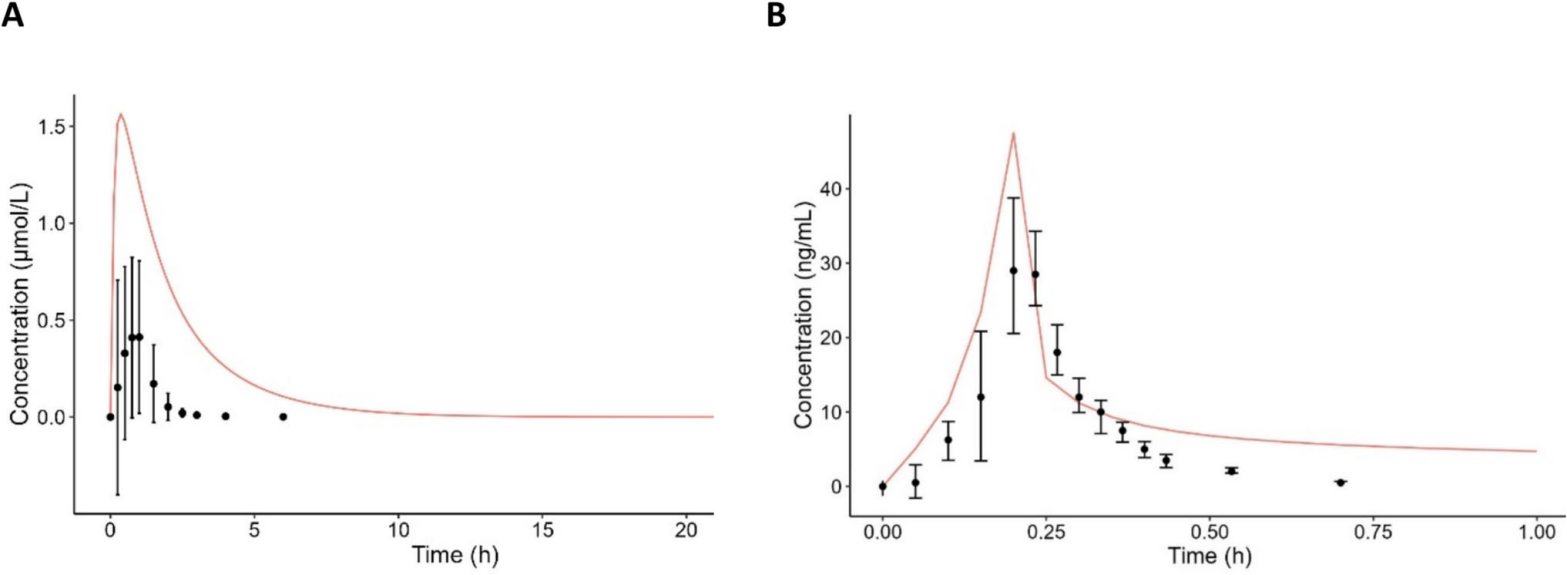
PBK model evaluation; **(A)** mean observed ± SD (black dots) (n = 88) and simulated (red line) plasma concentrations of tyramine in humans after an oral dose of 5.7 mg/kg bw (Rafehi et al. 2019); **(B)** mean observed ± SD (black dots) (n = 10) and simulated (red line) plasma concentrations of higenamine in humans after an intravenous infusion of 0.5, 1, 2, and 4 µg/kg bw with an infusion duration of 3 min per dose (Feng et al. 2012)

The predictive performance of the PBK model was evaluated by comparing the C_plasma venous_-time profiles of higenamine and tyramine with observed plasma concentrations in humans (Fig. 4). In the case of tyramine, there was a 3.78-fold over-prediction of the C_max plasma_ compared to the observed average (Fig. 4A). However, the modelled C_max plasma_ of 1.56 µmol/L was within the range of the observed concentrations in all 88 participants of the study, which was from 0.00729 to 2.02 µmol/L (Rafehi et al. 2019). A PBK model with an intravenous infusion of higenamine was built to evaluate the elimination phase of higenamine by the PBK model. The modelled plasma concentration of higenamine in the first part of the elimination phase, which occurred from 0.20 to 0.45 hours, was similar to the observed concentrations from the study of (Feng et al. 2012) (Fig. 4B). An overestimation by the PBK model can be seen at the final part of the elimination phase, since the observed average plasma concentration at t = 0.70 h is 0.5 ng/mL, while the modelled concentration 5.58 ng/mL is .

### Simulated internal concentrations

**Fig. 5:**
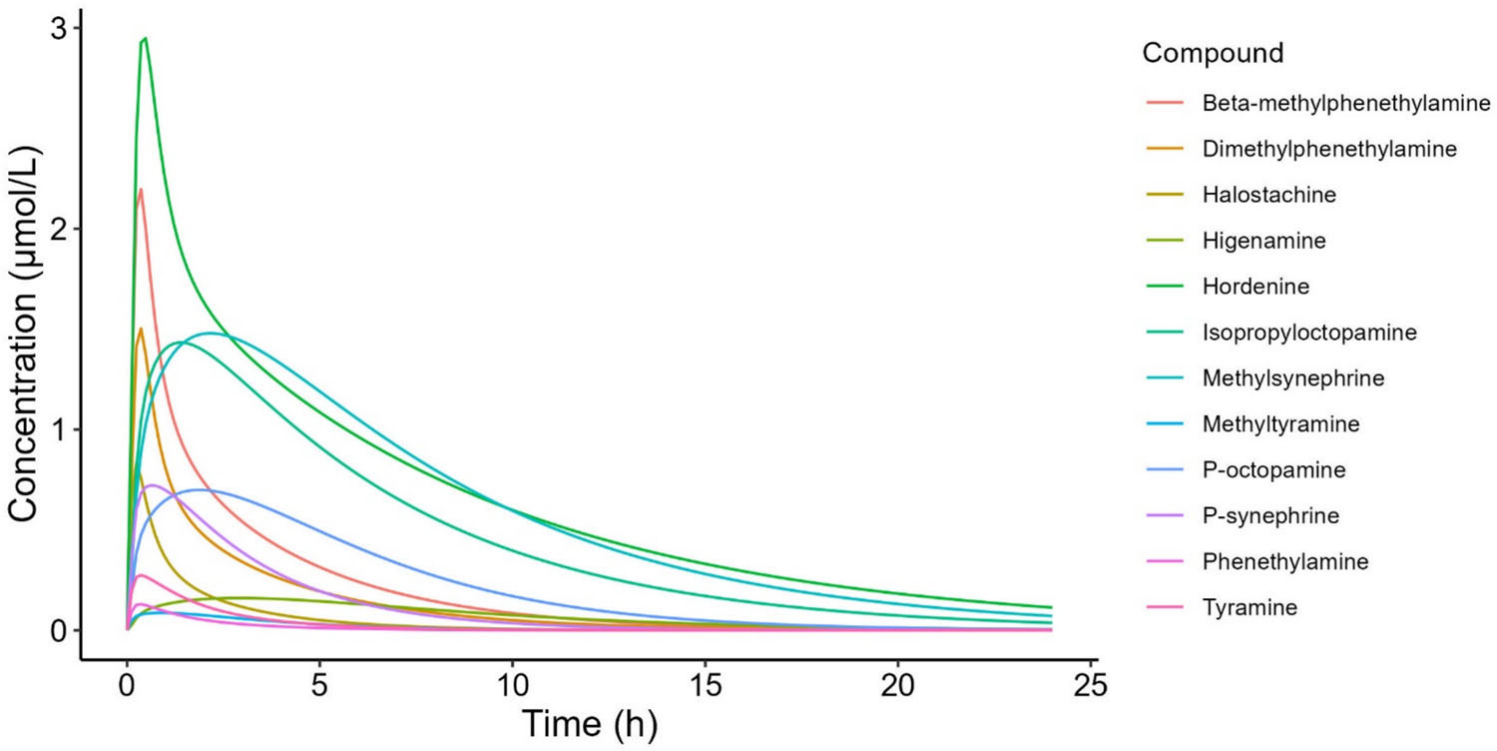
Simulated plasma concentration–time profiles of the studied phenethylamine analogues at an oral dose of 1 mg/kg bw

The developed PBK model with oral dosing was used to predict the plasma concentrations of PEA and its analogues after an oral dose of 1 mg/kg bw (Fig. 5). The predicted tissue:plasma partition coefficients that were used as input parameters are presented as supplementary data (InputParameters.csv). The C_max plasma venous_ of the studied compounds ranged from 0.0870 µM for methyltyramine to 2.95 µM for hordenine (Table 6).

**Table 6:** Simulated maximal plasma concentrations in the venous plasma compartment (C_max, plasma venous_) at an oral dose of 1 mg/kg bw

Compound	C_max, plasma venous_ (µM)
β-methylphenethylamine	2.20
Dimethylphenethylamine	1.50
Halostachine	0.832
Higenamine	0.161
Hordenine	2.95
Isopropyloctopamine	1.43
Methylsynephrine	1.48
Methyltyramine	0.0870
*p*-Octopamine	0.699
*p*-Synephrine	0.722
Phenethylamine	0.130
Tyramine	0.274

### Prediction of oral equivalent effect doses

The in vitro effect concentrations (EC_50_), for activation of the α_1A,_ α_1B,_ α_1D,_ α_2A,_ α_2B,_ β_1_ and β_2_ adrenergic receptor (ADR) activation and the activation of TAAR1, were obtained from our previous study (Pinckaers et al. 2024). These EC_50_ values were converted to equivalent effect doses (ED_50_) with the PBK modelling-based reverse dosimetry approach. The predicted ED_50_ values of PEA and its analogues for in vivo activation of the ADRs and TAAR1 are presented in Table 7. The predicted ED_50_ values for activation of ADRα_1A/B/D_ are within a range of 0.914 to 29.7 mg/kw bw. The ED_50_ values for activation of ADRα_2A_ by hordenine and *p*-synephrine are 234 and 139 mg/kg bw, respectively. Effective doses for activation of the ADRβ_1_ are the lowest of all studied receptors, with especially isopropyloctopamine and higenamine being very potent (ED_50_ = 0.0839 and 0.211 mg/kg bw, respectively). The effective doses for activation of TAAR1 range from 0.995 mg/kg bw for β-methylphenethylamine to 264 mg/kg bw for methyltyramine. Comparison of the predicted ED_50_ values with reported intake values, reveals that particularly higenamine, isopropyloctopamine, β-methylphenethylamine and *p*-synephrine exposure is in the same range or exceeds the predicted ED_50_ values (Table 7).

**Table 7:** Predicted ED_50_ values (in mg/kg bw) of phenethylamine and its analogues for ADRα_1A/B/D_, ADRα_2a_ ADRβ_1/2_ and TAAR1 based on PBK modelled maximal plasma concentrations (C_max plasma venous_) and reported daily doses or dose ranges (in mg/kg bw); compounds with a dash (-) did not activate the receptors in vitro at concentrations ≤ 300 µM. The reported daily intake is based on the detected amount in 1 food supplement dosage and is normalized to kg/bw based on an averaged weight individual of 70 kg

Compound	ADRα_1A_ ED_50_	ADRα_1B_ ED_50_	ADRα_1D_ ED_50_	ADRα_2A_ ED_50_	ADRβ_1_ ED_50_	ADRβ_2_ ED_50_	TAAR1 ED_50_	Reported daily dose (mg/kg bw)
β-methylphenethylamine	–	–	–	–	–	–	0.955	1.31–14.0 Cohen et al. (2021); Cohen et al. (2015b); Duiven et al. (2021)
Dimethylphenethylamine	–	4.07	5.60	–	–	–	14.0	0.214, 6.86 Duiven et al. (2021)
Halostachine	10.5	1.32	2.52	–	–	–	88.9	*not available*
Higenamine	–	–	–	–	0.211	2.92	6.09	0.44 1.37 Biesterbos et al. (2019); Cohen et al. (2021)
Hordenine	–	1.90	11.5	234	–	–	15.9	1.19 (Biesterbos et al. 2019)
Isopropyloctopamine	–	–	–	–	0.0839	–	1.26	0.557–0.990 Bovee et al. (2016); Cohen et al. (2021)
Methylsynephrine	29.7	–	–	–	16.9	–	–	0.00127 – 11.4 Pawar et al. (2020); Duiven et al. (2021)
Methyltyramine	–	–	–	–	–	–	264	0.0000142 – 0.371 Pawar et al. (2020)
*p*-Octopamine	15.7	5.58	1.72	–	7.87	–	65.8	0.001 – 1.86 Biesterbos et al. (2019) Pawar et al. (2020) Koh et al. (2021)
*p*-Synephrine	3.32	0.914	2.35	139	38.8	–	127	0.000129 – 2.29 Biesterbos et al. (2019) Pawar et al. (2020) Viana et al. (2016)
Phenethylamine	–	–	–	–	–	–	67.7	0.17 Biesterbos et al. (2019)
Tyramine	–	–	–	–	–	–	34.7	0.0000129 – 0.0157 Pawar et al. (2020) Koh et al. (2021)

**Fig. 6:**
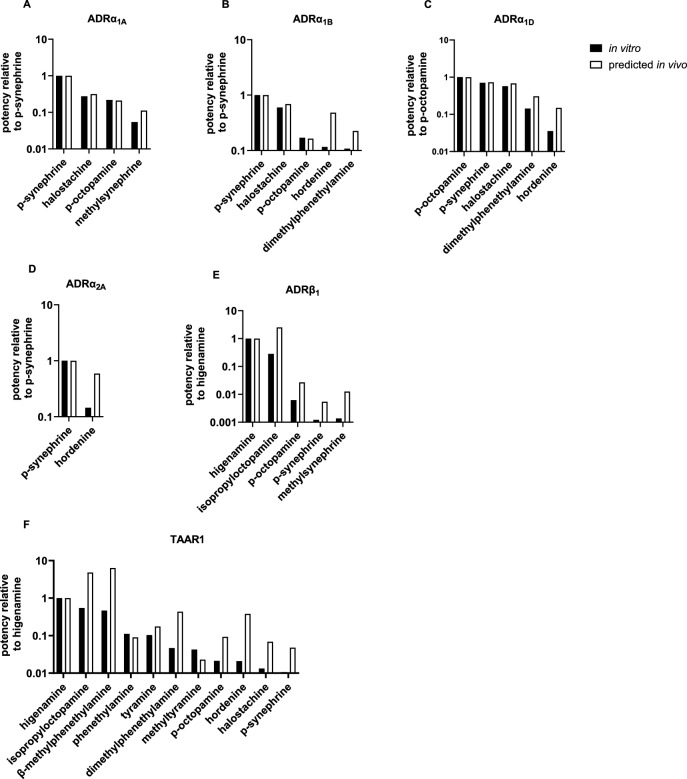
Relative potencies of the studied phenethylamine analogues that activated (**A**) ADRα_1A_, (**B**) ADRα_1B_, (**C**) ADRα_1D_, (**D**) ADRα_2A_, (**E**) ADRβ_1_ and (**F**) TAAR1; potencies are normalized to the most potent compound in the in vitro assay; the closed bars present the in vitro relative potencies and the open bars present the predicted in vivo potencies

**Fig. 7:**
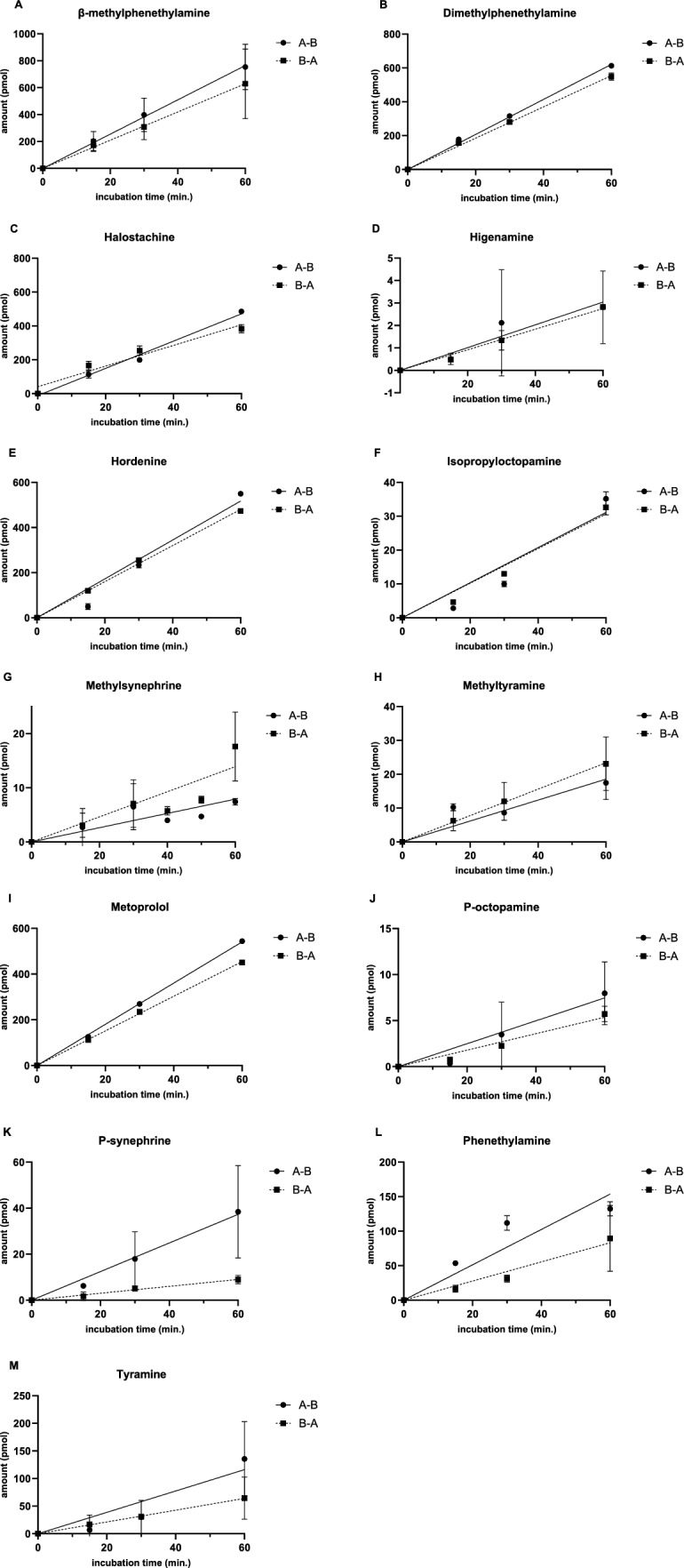
In vitro transport of Phenethylamine (analogues) and model compound metoprolol across Caco-2 cell layers at a starting concentration of 10 µM**; c**umulative amount of **(A)** β-methylphenethylamine, **(B)** dimethylphenethylamine, **(C)** halostachine, **(D)** higenamine, **(E)** hordenine, **(F)** isopropyloctopamine, **(G)** methylsynephrine, **(H)** methyltyramine, **(I)** metoprolol, **(J)** p-octopamine, **(K)** p-synephrine, **(L)** phenethylamine and **(M)** tyramine in the basolateral compartment (for A-B studies*) or in the apical compartment (for B-A studies**) during transport after apical (for A-B) or basolateral (for B-A) exposure to 10 µM of each compound; samples were taken from the receiver compartment (B for A-B and A for B-A studies) after 15, 30 and 60 min; the amount of compound (in pmol) was quantified using LC–MS/MS; data are presented as mean values ± SD from duplicate wells. * A-B studies: transport direction from the apical to the basolateral compartment was studied by adding the compound to the apical side at the start of the experiment. ** B-A studies: transport direction from the basolateral to the apical compartment was studied by adding the compound to the basolateral side at the start of the experiment

**Fig. 8:**
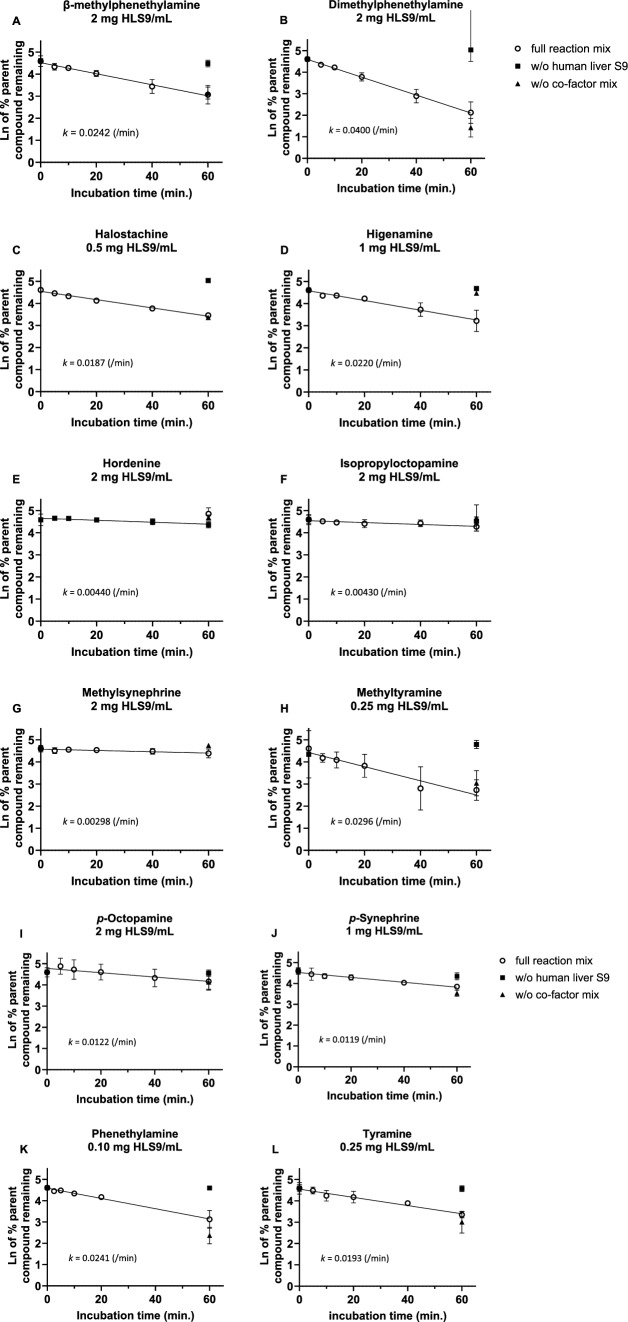
Compound depletion of a starting concentration of 1 µM of **(A)** β-methylphenethylamine, **(B)** dimethylphenethylamine, **(C)** halostachine, **(D)** higenamine, **(E)** hordenine, **(F)** isopropyloctopamine, **(G)** methylsynephrine, **(H)** methyltyramine, **(I)** p-octopamine, **(J)** p-synephrine, **(K)** phenethylamine and **(L)** tyramine in incubations with human liver S9 (HSL9) and co-factor mix (= full reaction mix; **dots**), in incubations with co-factor mix and without HLS9 (= w/o human liver S9; **squares**) and in incubations with HLS9 and without co-factor mix (= w/o co-factor mix; **triangles**); data points (± SD) are shown as the natural logarithm (Ln) of the percentage of the parent compound at time point zero; K represents the elimination rate constant (/min) derived from the slope of compound depletion in full reaction mix incubations

**Fig. 9:**
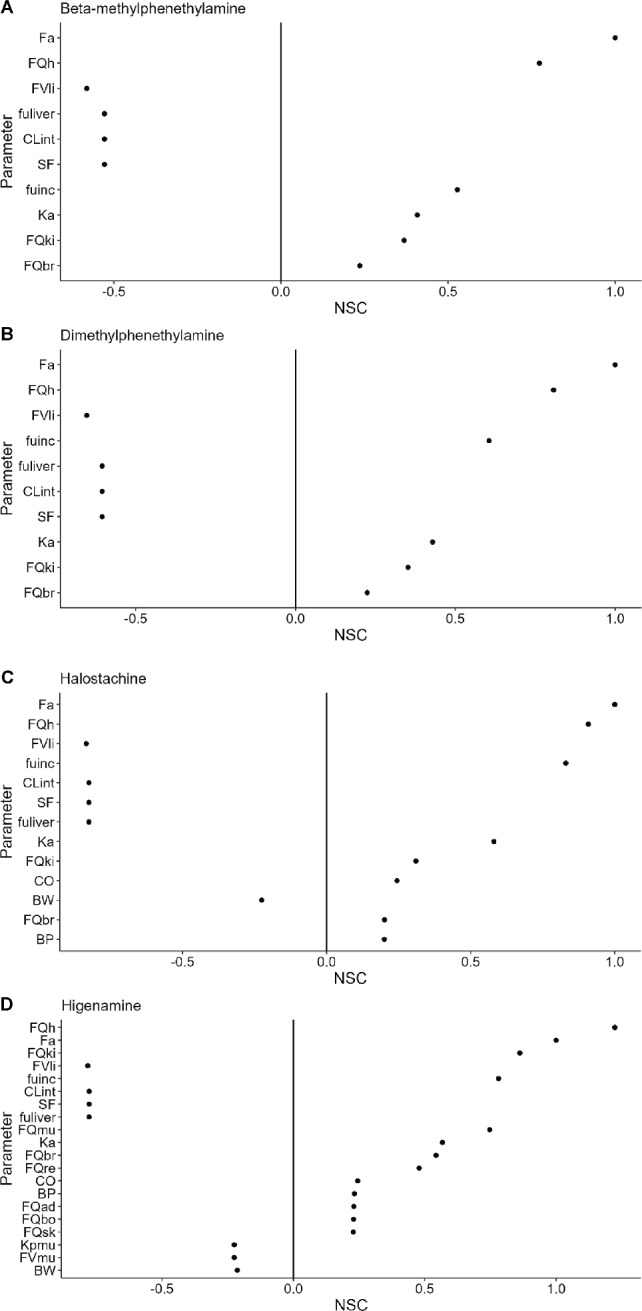

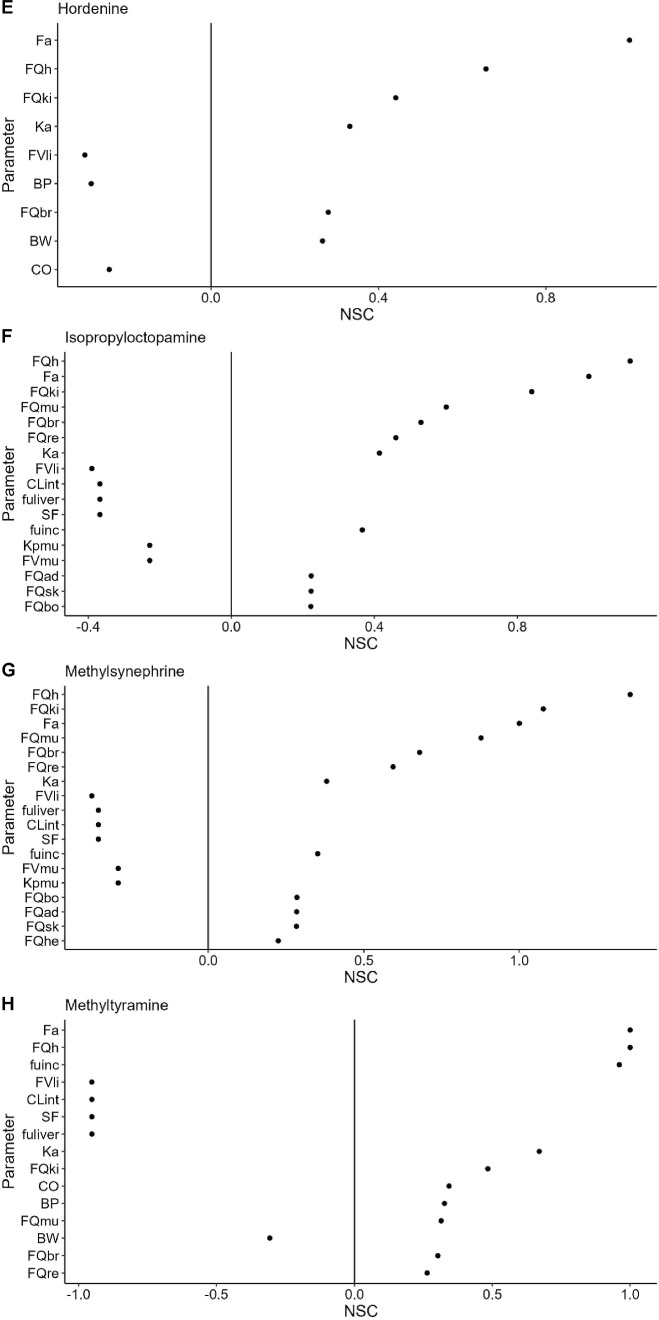

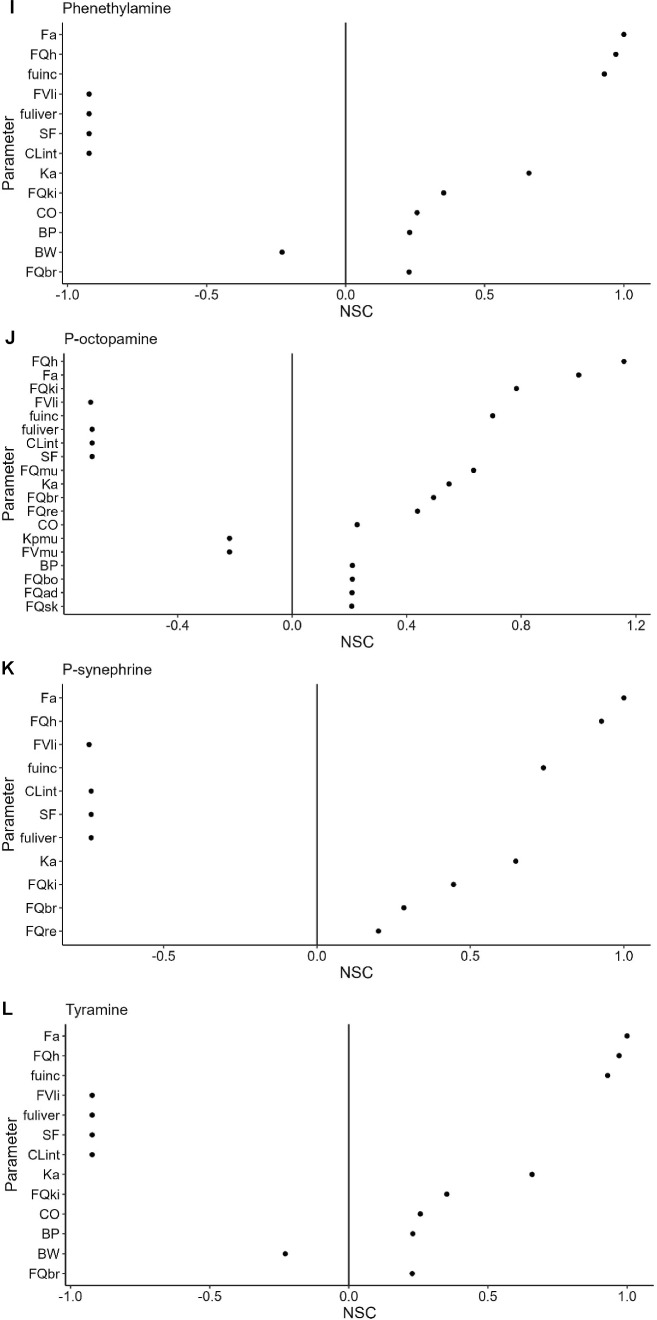
Normalised sensitivity coefficients (NSCs) of the C_max plasma venous_ simulations to the critical input parameters for all studied phenethylamine (PEA) analogues at an oral dose of 1 mg/kg bw; (A) β-methylphenethylamine, (B) dimethylphenethylamine, (C) halostachine, (D) higenamine, (E) hordenine, (F) isopropyloctopamine, (G) methylsynephrine, (H) methyltyramine, (I) phenethylamine, (J) p-octopamine, (K) p-synephrine and (L) tyramine. FQre = fractional tissue blood flow rest of body, FQte = fractional testes blood, FQsp = fractional spleen blood flow, FQmu = fractional muscle blood flow, FQh = fractional hepatic blood flow, FQki = fractional kidney blood flow, FQhe = fractional heart blood flow, FQgu = fractional gut blood flow, FQbr = fractional brain blood flow, FQbo = fractional bone blood flow, FQad = fractional adipose tissue blood flow, CO = cardiac output, FVre = fractional volume rest of body, FVpl = fractional volume plasma, FVar = fractional volume arterial blood, FVve = fractional volume venous blood, FVte = fractional volume testes, FVsp = fractional volume spleen, FVsk = fractional volume skin, FVmu = fractional volume muscle, FVlu = fractional volume lung, FVli = fractional volume liver, FVki = fractional volume kidney, FVhe = fractional volume heart, FVgu = fractional volume gut, FVbr = fractional volume brain, FVbo = fractional volume bone, FVad = fractional volume adipose tissue, BW = body weight, BP = blood/plasma ratio, fuliver = fraction unbound liver, fup = fraction unbound in plasma, CLint = intrinsic clearance, fuinc = fraction unbound in human liver S9 incubation, SF = scaling factor of human liver S9 to a whole human liver, Kpre = rest of body:plasma partition coefficient, Kpte = testes:plasma partition coefficient, Kpsp = spleen:plasma partition coefficient, Kpsk = skin:plasma partition coefficient, Kpmu = muscle:plasma partition coefficient, Kplu = lung:plasma partition coefficient, Kpli = liver:plasma partition coefficient, Kpki = kidney:plasma partition coefficient, Kphe = heart:plasma partition coefficient, Kpgu = gut:plasma partition coefficient, Kpbr = brain:plasma partition coefficient, Kpbo = bone:plasma partition coefficient, Kpad = adipose:plasma partition coefficient, fa = fraction absorbed, ka = absorption rate constant

In the next step, the predicted ED_50_ values as shown in Table 7, of all the studied PEA analogues were related to the most potent compound in the in vitro receptor activation assays which was determined previously (Pinckaers et al. 2024). For activation of ADRα_1A_, ADRα_1B_ and ADRα_2A_, *p*-synephrine was the most potent compound in the in vitro assays. After applying the kinetics in this study, the relative difference in potency between *p*-synephrine and the other agonists became smaller (Fig. 6A,B,D). For the activation of ADR_1D_ a similar trend was seen, but then for the most potent agonist p-octopamine (Fig. 6C). The results of the in vitro ADRβ_1_ activation assays showed that higenamine was 3.5 times more potent than isopropyloctopamine (Fig. 6E). However, after simulating the plasma concentrations of these compounds, the relative potency of these compounds changed. The predicted equivalent oral dose for isopropyloctopamine was 2.5 times lower than the dose of higenamine needed to reach the internal effect concentration, as a result of faster intestinal uptake (Table 4) and slower hepatic metabolic of isopropyloctopamine (Table 5). This change in relative potency after applying the kinetics was also observed for the activation of TAAR1. Higenamine was the most potent agonist in the in vitro TAAR1 assays. However, the doses of β-methylphenethylamine and isopropyloctopamine needed to reach internal effect concentrations were 6.4 and 4.8 times lower than the dose of higenamine (Fig. 6F), because of the higher rate and amount of intestinal absorption (Table 4) and the lower hepatic metabolic clearance rate of β-methylphenethylamine and isopropyloctopamine (Table 5).

## Discussion

The aim of the present study was to predict oral effective doses (ED_50_) for humans of phenethylamine (PEA) and its analogues, to prioritize these compounds for further risk assessment. The studied PEAs have previously been shown to activate human ADRs and TAAR1 in vitro (Pinckaers et al. 2024), which implies that these compounds could affect (in)directly the cardiovascular system in vivo. Plasma concentrations of the PEAs were predicted using a generic PBK model (Jones and Rowland-Yeo 2013) and were linked to in vitro EC_50_ values for activation of ADRα_1A/B/D_, ADRα_2A_, ADRβ_1/2_ and TAAR1. Consequently, in vivo ED_50_ values were predicted by applying the reverse dosimetry approach. The predicted ED_50_ values of the studied PEAs for activation of ADRα_1A/B/D_, ADRα_2A_, ADRβ_1_ and TAAR1 were within a range of 0.914 to 29.7 mg/kg body weight (bw), 139 to 234 mg/kg bw, 0.0839 to 38.8 mg/kg bw and 0.995 to 264 mg/kg bw, respectively.

**Fig. 10:**
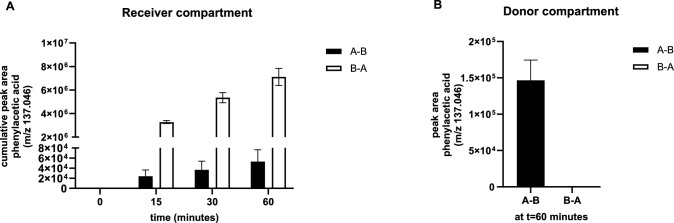
LC–MS analysis of the deaminated product of phenethylamine: phenylacetic acid (m/z 137.046) in medium collected from Caco-2 cells exposed to 10 µM phenethylamine; (A) cumulative peak area of phenylacetic acid in medium collected from the receiver compartment (= basolateral compartment in A-B and apical compartment in B-A studies) after 0, 15, 30 and 60 min of exposure; (B) phenylacetic acid in medium collected from the donor compartment (= apical compartment in A-B and basolateral compartment in B-A studies) after 60 min of exposure; data are presented as average peak area of m/z 137.046 from two different wells

To predict plasma concentrations of the PEA analogues with the PBK model, information about intestinal absorption and hepatic metabolic clearance for each of the compounds was required. These input parameters were extrapolated from experimentally collected in vitro data in the current study. Using transport studies with Caco-2 cells, the intestinal uptake rate and the intestinal fraction absorbed by the PEA analogues were extrapolated. Unlike the studied analogues, PEA itself had a poor recovery of 15.7% in the Caco-2 transport studies when it was added to the apical side of the cell culture system, whereas the recovery in the experiments studying the opposite transport direction (i.e. from the basolateral to the apical compartment) was 103%. This suggests that PEA was metabolized by Caco-2 cells, which was confirmed by the presence of the molecular ion of the deaminated product of PEA, phenylacetic acid, in medium collected from Caco-2 cells upon PEA exposure (Fig. 10 in the appendix). It has been previously shown that PEA is a substrate of monoamine oxidase B (MAO-B) that deaminates amines and that this enzyme is active in Caco-2 cells (Obata et al. 2022; Vieira-Coelho et al. 1999) (Iwasa et al. 2003). Although some of the other studied PEAs are also known MAO(-A/B) substrates (e.g. tyramine and *p*-synephrine) (Balsa et al. 1989; Oguchi et al. 1981) (Suzuki et al. 1979), their metabolic breakdown by Caco-2 cells was not observed in this study. This could be explained by the higher affinity that PEA has for MAO compared to other PEA analogues (Balsa et al. 1989; Oguchi et al. 1981). In addition, our study shows that the apparent intrinsic clearance is the highest for PEA, which is approximately 2 to 112 times higher than the clearance of its analogues (Table 5), which might explain the poor recovery of PEA, and not its analogues, in Caco-2 transport studies.

The hepatic metabolic clearance was extrapolated from in vitro studies with HLS9. All PEA analogues, except higenamine, were metabolized for 34.7 to 95.5% of the total metabolic clearance, in the absence of the co-factor mix that enabled oxidation, reduction, hydrolysis, glucuronidation, sulfation and methylation. This indicates that those compounds were metabolized by enzymes that did not need externally added co-factors, such as MAO.

The potential effects of the PEA metabolites and its analogues were not included in the current study, as this was out of our scope. Interestingly, for some of the studied PEAs it is known that they can be transformed into bioactive metabolites. For example, tyramine can be metabolized by the liver into *p*-octopamine and into dopamine (Faraj et al. 1979; Hiroi et al. 1998). Tyramine does not activate ADRs directly, whereas *p*-octopamine can activate α_1_ and β_1_ ADRs. Furthermore, *p*-octopamine and *p*-synephrine were also found to be metabolized into noradrenaline and adrenaline, respectively, by CYP2D6 in human liver microsomes (Hiroi et al. 1998). Hence, besides the suggested effects after the oral consumption of the studied PEAs, some of the metabolites could have additional pharmacological effects when metabolized into other bioactive molecules. The predicted intrinsic metabolic clearance of reference compound tyramine (841 L/hr), scaled from the in vitro HLS9 incubations in this study (Table 5), was in the same range as the metabolic clearance observed in humans (792 L/hr) (Faraj et al. 1979). This highlights the relevance and predictive value of incorporating in vitro hepatic clearance in the PBK model. The adequacy of the in vitro hepatic clearance was also shown by comparing the predicted higenamine plasma elimination and the observed plasma elimination, which were in the same range (Fig. 4B).

The predicted intestinal and hepatic kinetic data were integrated into the PBK model, after which the plasma concentrations were simulated. The performance of the PBK model was evaluated by comparing the predicted plasma concentration–time profiles of tyramine with observed concentrations in humans (Fig. 4A). There was a 3.78-fold over-prediction of the C_max plasma_ of tyramine compared to the observed average, which may be explained by an overestimation of the intestinal absorption or an underestimation of the volume of distribution (Punt et al. 2022). Overestimations of C_max plasma_ values are frequently observed when using generic PBK models, which might be explained by their lack of extrahepatic metabolism or active efflux transport (Punt et al. 2022). Interestingly, the modelled C_max plasma_ of 1.56 µmol/L was still within the range of the observed concentrations (i.e. between 0.00729 and 2.02 µmol/L) of all 88 participants of the study. This highlights that there is high variability between humans in plasma concentrations after an oral dose of tyramine and that the generic PBK model used in our study also covers individuals with higher plasma concentrations. With regards to risk assessment of purposes, this is especially important as individuals at higher risk for the toxicological effects of these compounds should be protected.

In the current study, a generic PBK model with standard physiological parameters was used to predict plasma concentrations and in vivo potencies of the PEA analogues (Jones and Rowland-Yeo 2013). The cardiac output (CO) in the used PBK model was 108.33 mL/s, which represents the physiology of a resting individual (Brown et al. 1997; Joyner and Casey 2015). Physical exercise is one of the factors that influences CO and tissue perfusion, and thereby also the kinetics. The CO can increase by 29%, 52% and 362% during light, moderate and heavy exercise, respectively (Brown et al. 1997). On the contrary, blood flow to the liver and intestinal tissues is decreased to compensate for an increased blood flow to (cardiac) muscle and bone tissue. Exercise could therefore reduce the C_max plasma_ but with a retained AUC, as a result of a slower hepatic metabolic breakdown. PEA and its analogues are often used as pre-workout supplements to allow intestinal absorption and effective plasma concentrations before starting the workout. This implies that the intestinal absorption occurs during resting conditions, but that the hepatic metabolic breakdown takes place during exercise. The impaired liver perfusion during exercise decreases the hepatic metabolic breakdown, which leads to plasma concentrations that could be at an effective level for a longer time compared to situations during rest. This could increase the exposure time and the associated risk of pharmacological effects and should be taken into account in future PBK modelling and risk assessment studies.

The predicted ED_50_ values in this study are in the same range as reported daily doses in food supplements for some of the studied PEAs (Table 7), which implies that cardiovascular effects could be expected in vivo. Strikingly, the reported daily intake doses of isopropyloctopamine were about ten times higher than the predicted dose needed to reach plasma concentrations similar to the EC_50_ to activate ADRβ_1_, suggesting that intake may lead to ADRβ_1_ mediated effects, such as tachycardia (Alhayek and Preuss 2024). Adverse cardiac effects were indeed previously linked to the use of a food supplement containing isopropyloctopamine (Bovee et al. 2016). The use of this food supplement was associated with multiple hospitalisations and even in one case, the patient died as a result of a cardiac arrest. Also for other PEA analogues, reported daily intake doses were found to be in the same range or higher than predicted ED_50_ values needed to reach plasma concentrations to activate ADRα_1A/B_ (*p*-synephrine), ADRα_1D_ (*p*-synephrine, *p*-octopamine), ADRβ_1_ (higenamine) (Hudzik et al. 2020) and TAAR1 (β-methylphenethylamine, isopropyloctopamine) (Table 7). These results imply that users of food supplements containing these pharmacologically active substances may expect sympathetic activation and cardiovascular effects and could thus be at risk for (severe) adverse health effects. Hence, these compounds should be considered as high-priority compounds for further risk assessment. Our study furthermore highlights the importance of including kinetics in compound prioritization for risk assessment purposes, since the relative potencies changed for the activation of ADRβ_1_ and TAAR1 when using the PBK modelling-based reverse dosimetry approach compared to prioritization based on only in vitro potencies (Fig. 6).

The safety regulation of food supplements containing pharmacologically active compounds in The Netherlands, and other European countries, is rather complex (Biesterbos et al. 2019; Czepielewska et al. 2018). In case the food supplement contains pharmacologically active compounds that (1) are not classified as medicinal products, (2) do not fall under the Commodity law herbal preparations & Regulation (EC) No 1925/2006, (3) are not classified as novel food (Regulation EU) 2015/2283 and (4) are not proven to be unsafe (i.e. the General food law: food cannot be for sale when it is not safe, Regulation (EC) No 178/2002), it stays freely available on the market (Biesterbos et al. 2019). This indicates that, when there is no information available concerning pharmacological and toxicological effects, food supplements with pharmacologically active compounds can stay on the market until proven to pose a risk to human health. The Office of Risk Assessment & Research of the Netherlands Food and Consumer Product Safety Authority performs those risk assessments in The Netherlands. As the list of compounds in food supplements with an unknown safety profile is growing (Czepielewska et al. 2018) and since there is no legal requirement to prove food safety before entering the market (Biesterbos et al. 2019), there is a high need for prioritization methods that can accelerate human health risk assessment. Our study shows that the use of QIVIVE of adrenergic and TAAR1 potencies using a generic PBK model (with minimal required input data to describe passive intestinal uptake, liver metabolism, partition coefficients, fraction unbound in plasma, and blood:plasma ratio) can serve as an efficient prioritization method for a whole set of chemical analogues. In case new chemical analogues with an unknown safety profile are detected in food supplements, the above-described case study can easily be expanded and applied to the particular compounds. Generic PBK models can, however, not be applied for all chemicals, since the predictive performance cannot be guaranteed. However, the use of PBK models for groups of chemical analogues offers more reliable QIVIVEs (Najjar et al. 2022).

In conclusion, our study demonstrates the applicability of QIVIVE and PBK modelling as NAMs to prioritize large sets of compounds for risk assessment. Our findings suggest that the consumption of food supplements containing higenamine, isopropyloctopamine, β-methylphenethylamine and *p*-synephrine could result in effective plasma concentrations to activate ADRs or TAAR1, that (in)directly affect the cardiovascular system. These PEA analogues should therefore be considered as high priority compounds for further risk assessment.

## Electronic supplementary material

Below is the link to the electronic supplementary material.

Supplementary file1 (PDF 43 kb)

Supplementary file2 (PDF 35 kb)

## Appendix

**Table 8:** *Mean apparent permeability (P*_*app*_ ± *SD) values, recovery rates* ± *SD and efflux ratios derived from the* in vitro *Caco-2 permeability assays studying apical to basolateral (A-B) and basolateral to apical (B-A) transport*

*Compound*	*A-B* *P*_*app*_* (*× *10*^*–6*^* cm/s)*	*Recovery (%)*	*B-A* *P*_*app*_* (*× *10*^*–6*^* cm/s)*	*Recovery (%)*	*Efflux ratio*
β-methylphenethylamine	15.8 ± 1.47	80.9 ± 3.27	12.1 ± 2.02	89.3 ± 4.46	0.766
Dimethylphenethylamine	14.7 ± 0.244	76.7 ± 0.06	13.1 ± 0.510	93.3 ± 4.81	0.891
Halostachine	13.1 ± 0.144	84.0 ± 3.18	10.3 ± 0.649	93.9 ± 0.0289	0.786
Higenamine	0.0610 ± 0.0351	104 ± 0.35	0.0612 ± 0.00186	105 ± 1.06	1.00
Hordenine	13.0 ± 0.192	96.7 ± 1.27	11.2 ± 0.220	107 ± 0.0262	0.862
Isopropyloctopamine	0.703 ± 0.0410	79.8 ± 2.19	0.652 ± 0.0444	76.5 ± 1.27	0.927
Methylsynephrine	0.353 ± 0.215	114 ± 8.39	0.390 ± 0.202	102 ± 11.7	1.10
Methyltyramine	0.344 ± 0.0965	87.1 ± 9.14	0.455 ± 0.156	85.2 ± 10.4	1.32
Metoprolol	11.2 ± 0.197	103 ± 2.30	9.25 ± 0.170	103 ± 8.60	0.826
*p*-Octopamine	0.164 ± 0.0701	98.9 ± 1.77	0.117 ± 0.0171	109 ± 15.0	0.713
*p*-Synephrine	0.951 ± 0.497	109 ± 1.48	0.220 ± 0.0453	100 ± 4.89	0.231
Phenethylamine	2.88 ± 0.220	15.7 ± 1.51	1.95 ± 1.04	103 ± 3.36	0.677
Tyramine	2.38 ± 1.186	114 ± 11.2	1.13 ± 0.670	84.0 ± 6.25	0.475

**Table 9:** P_app A-B_ values of metoprolol in Caco-2 permeability assays as reported in literature (average = 40.7 × 10^–6^ cm/sec ± 35.5; coefficient of variation = 87.3%; n = 20)

P_app_ × 10^–6^ (cm/sec)	References
8.19	Lee et al. (2017)
31.8	Alsenz and Haenel (2003)
140	Irvine et al. (1999)
33.2	Li et al. (2007)
23.7	Zhu et al. (2002)
17.7	Skolnik et al. (2010)
2.30	Kerns et al. (2004)
40.0	Teksin et al. (2010)
22.5	Incecayir et al. (2013)
36.9	Yang et al. (2007)
21.2	Vaithianathan et al. (2015)
60.1	Punt et al. (2021a)
29.0	Galkin et al. (2008)
18.8	Hilgendorf et al. (2000)
23.7	Yazdanian et al. (1998)
27.0	Artursson and Karlsson (1991)
91.9	Palm et al. (1996)
41.9	Lentz et al. (2000)
116	Neuhoff et al. (2003)
28.1	Gertz et al. (2010)
