# Supplementary material for: Quantitative in vitro-to-in vivo extrapolation of human adrenergic and trace amine-associated receptor 1 potencies of pre-workout supplement ingredients using physiologically based kinetic modelling-based reverse dosimetry
Source: Arch Toxicol. 2025 Apr 3;99(5):1999–2021. doi: 10.1007/s00204-025-03992-7 (PMC12085399; doi:10.1007/s00204-025-03992-7)
Supplement: Supplementary file 1 — Supplementary file1 (PDF 43 kb) [file 204_2025_3992_MOESM1_ESM.pdf]

Quantitative in vitro-to-in vivo extrapolation of human adrenergic and trace amine associated receptor 1 potencies of pre-workout supplement ingredients using physiologically based kinetic modeling-based reverse dosimetry

Archives of Toxicology

Nicole E.T. Pinckaers [1,2,*], W. Matthijs Blankesteijn [1,3], Anastasiya Mircheva [1,2], Ans Punt [4], Antoon Opperhuizen [1,2,5], Frederik-Jan van Schooten [1,2], Misha Vrolijk [1,2]

[1] *Department of Pharmacology and Toxicology, Maastricht University, Maastricht, The Netherlands*
[2] *Institute of Nutrition and Translational Research in Metabolism (NUTRIM), Maastricht University, Maastricht, The Netherlands*
[3] *Cardiovascular Research Institute Maastricht (CARIM), Maastricht University, Maastricht, The Netherlands*
[4] *Ans Punt Computational Toxicology, Arnhem, The Netherlands*
[5] *Office for Risk Assessment and Research, Netherlands Food and Consumer Product Safety Authority (NVWA), Utrecht, The Netherlands*

* Corresponding author: n.pinckaers@maastrichtuniversity.nl

```
## Original code by Jones and Rowland-Yeo 2013 in Berkeley Madonna.
#https://doi.org/10.1038/psp.2013.41
## Converted to R by Ans Punt, March 2023, with the following modifications:
#- Conversion of the model code from Berkeley Madonna to R, solving the differential equations with
the R rxode2 package.
#- Cliverfree defined as Cliver/Kpli*Fupinstead of Cliver*Fup.
#- Fraction absorbed (F) accounted for in the initial setting of the dose rather than the differential
equation for oral absorption (the latter would be the rate of absorption and          not the fraction that is
absorbed).
#- Renal clearance (urinary excretion) simulated as GFR times the free plasma concentration.(Within
the model of Jones and Rowland-Yeo(2013), renal clearance is described as CLrenal*Ckidneyfreeand
it is not specified whether this renal clearance corresponds to urinary excretion or metabolic
clearance.)
#- Conversion of plasma concentration to nM as output of the model.
#- Kpre(tissue:partion coefficient rest body) set equal to the muscle partition coefficient.
#- Mass balance equations added
#- Removal of Vrb, Vplas_ven, and Vplas_art as these are not as such used in the model


#  set path to Rtools if needed
path <- Sys.getenv("PATH")
path <- c("C:/rtools/bin", "C:/rtools/mingw64/bin", path)
path <- paste(path,collapse=";")
Sys.setenv(PATH=path)
Sys.getenv("PATH")

# LIBRARIES #####
library(rxode2)
library(tidyverse)
```

```
# PBK MODEL #####
PBK_model <- rxode2({

## SPECIES SPECIFIC PARAMETERS #####

### Fractional tissue volumes #####
  # values for the fractional tissue volumes are derived from the parameter input file
  BW = BW                    # BW (kg)
  FVad = FVad                # adipose
  FVbo = FVbo                # bone
  FVbr = FVbr                # brain
  FVgu = FVgu                # gut
  FVhe = FVhe                # heart
  FVki = FVki                # kidney
  FVli = FVli                # liver
  FVlu = FVlu                # lung
  FVmu = FVmu                # muscle
  FVsk = FVsk                # skin
  FVsp = FVsp                # spleen
  FVte = FVte                # testes
  FVve = FVve                # venous
  FVar = FVar                # arterial
  FVpl = FVpl                # plasma
  FVre = FVre                # rest of body

### Total tissue volumes - L #####
  Vad = BW*FVad
  Vbo = BW*FVbo
  Vbr = BW*FVbr
  Vgu = BW*FVgu
  Vhe = BW*FVhe
  Vki = BW*FVki
  Vli = BW*FVli
  Vlu = BW*FVlu
  Vmu = BW*FVmu
  Vsk = BW*FVsk
  Vsp = BW*FVsp
  Vte = BW*FVte
  Vve = BW*FVve
  Var = BW*FVar
  Vpl = BW*FVpl
  Vre = BW*FVre

### Fractional tissue blood flows #####
  FQad = FQad                # adipose
  FQbo = FQbo                # bone
  FQbr = FQbr                # brain
  FQgu = FQgu                # gut
  FQhe = FQhe                # heart
  FQki = FQki                # kidney
  FQh = FQh                  # hepatic (venous side)
  FQlu = FQlu                # lung
  FQmu = FQmu                # muscle
  FQsk = FQsk                # skin
  FQsp = FQsp                # spleen
```

```
  FQte = FQte               # testes
  FQre = FQre               # rest of body

### Total tissue blood flows - L/hr #####
  CO = CO                          #cardiac output (ml/s)
  QC = CO/1000*60*60               #cardiac output (L/hr)
  Qad = QC*FQad
  Qbo = QC*FQbo
  Qbr = QC*FQbr
  Qgu = QC*FQgu
  Qhe = QC*FQhe
  Qki = QC*FQki
  Qh = QC*FQh
  Qha = QC*FQh - QC*FQgu - QC*FQsp
  Qlu = QC*FQlu
  Qmu = QC*FQmu
  Qsk = QC*FQsk
  Qsp = QC*FQsp
  Qte = QC*FQte
  Qre = QC*FQre

## COMPOUND SPECIFIC PARAMETERS #####
### Absorption #####
  Ka = Ka                   #Ka (hr-1)
  Fa = Fa                   #fraction absorbed

### Tissue to plasma partition coefficients ######
  Kpad = Kpad               # adipose
  Kpbo = Kpbo               # bone
  Kpbr = Kpbr               # brain
  Kpgu = Kpgu               # gut
  Kphe = Kphe               # heart
  Kpki = Kpki               # kidney
  Kpli = Kpli               # liver
  Kplu = Kplu               # lung
  Kpmu = Kpmu               # muscle
  Kpsk = Kpsk               # skin
  Kpsp = Kpsp               # spleen
  Kpte = Kpte               # testes/gonads
  Kpre = Kpre               # rest of body

### Clearances #####
#Hepatic metabolic clearance
  SF = SF                   #1X10^6 cell/g liver or mg S9 or microsomal protein/g liver
  fuinc = fuinc             #fraction unbound in the in vitro incubation
  CLint = CLint             #in vitro intrinsic hepatic metabolic clearance (ul/min/10^6) cell
  CLmet = (CLint/fuinc)*SF*Vli*60/1000 #CLint scaled (L/hr)

### Passive renal clearance #####
  GFR = 7                   #glomerular filtration rate (GFR) (L/hr)

### In vitro/in silico binding data #####
  fup = fup                 #fraction unbound in plasma
  BP = BP                   #blood to plasma ratio
  fuliver = fuliver
```

```
## CALCULATION OF TOTAL CONCENTRATIONS - mg/L #####
  Cadipose = Aad/Vad
  Cbone = Abo/Vbo
  Cbrain = Abr/Vbr
  Cgut = Agu/Vgu
  Cheart = Ahe/Vhe
  Ckidney = Aki/Vki
  Cliver = Ali/Vli
  Clung = Alu/Vlu
  Cmuscle = Amu/Vmu
  Cskin = Ask/Vsk
  Cspleen = Asp/Vsp
  Ctestes = Ate/Vte
  Cvenous = Ave/Vve
  Carterial = Aar/Var
  Crest = Are/Vre
  Cplasmavenous = Cvenous/BP
  Cplasmavenous_uM = Cplasmavenous/MW*1000
  Cliverfree = Cliver/Kpli*fuliver
  Cplasmavenousfree = Cplasmavenous*fup

# DIFFERENTIAL EQUATIONS BODY ####
  d/dt(D)= -Ka*D
  d/dt(Dabs) = Ka*D*Fa*BW
  d/dt(Aad) = Qad*(Carterial - Cadipose/Kpad*BP)

  d/dt(Abo) = Qbo*(Carterial - Cbone/Kpbo*BP)

  d/dt(Abr) = Qbr*(Carterial - Cbrain/Kpbr*BP)

  d/dt(Agu) = d/dt(Dabs) + Qgu*(Carterial - Cgut/Kpgu*BP)
  d/dt(Ahe) = Qhe*(Carterial - Cheart/Kphe*BP)

  d/dt(Aki) = Qki*(Carterial - Ckidney/Kpki*BP) - GFR*Cplasmavenousfree

  d/dt(Ali) = Qha*Carterial + Qgu*(Cgut/Kpgu*BP) + Qsp*(Cspleen/Kpsp*BP) - Qh*(Cliver/Kpli*BP) -
Cliverfree*CLmet
  d/dt(Alu) = Qlu*Cvenous - Qlu*(Clung/Kplu*BP)
  d/dt(Amu) = Qmu*(Carterial - Cmuscle/Kpmu*BP)
  d/dt(Ask) = Qsk*(Carterial - Cskin/Kpsk*BP)
  d/dt(Asp) = Qsp*(Carterial - Cspleen/Kpsp*BP)
  d/dt(Ate) = Qte*(Carterial - Ctestes/Kpte*BP)
  d/dt(Ave) = Qad*(Cadipose/Kpad*BP) + Qbo*(Cbone/Kpbo*BP) + Qbr*(Cbrain/Kpbr*BP) +
Qhe*(Cheart/Kphe*BP) + Qki*(Ckidney/Kpki*BP) +
            Qh*(Cliver/Kpli*BP)  + Qmu*(Cmuscle/Kpmu*BP) + Qsk*(Cskin/Kpsk*BP) +
Qte*(Ctestes/Kpte*BP) + Qre*(Crest/Kpre*BP) - Qlu*Cvenous

  d/dt(Aar) = Qlu*(Clung/Kplu*BP) - Qlu*Carterial
  d/dt(Are) = Qre*(Carterial - Crest/Kpre*BP)
  #{Defining amount metabolitezed and cleared by the kidney for the mass balance equation}
  d/dt(AliClearance) =  Cliverfree*CLmet
  d/dt(AkiClearance) =  GFR*Cplasmavenousfree
  #{Mass balance}
  d/dt(MassBal) = Dabs -(Aad+Abo+Abr+Agu+Ahe+Aki+Ali+Alu+
                      Amu+Ask+Asp+Ate+Ave+Aar+Are+
```

```
                        AliClearance+AkiClearance)
  })

# RUN PBK MODEL #####
odose = 1 #mg/kg bw
parameters<-read.csv("InputParamteres.csv", sep = ";")
ev <- et(amount.units = "mg", time.units = "h") %>%
  #dose in mg/kgBW in the model this is multiplied by xFaxBW = dose in mg
  et(dose = odose, nbr.doses = 1, dosing.interval = 24, cmt = "D")
inits <- c(0,0,0,0,0,0,0,0,0,0,0,0,0,0,0,0,0,0,0,0,0)

#Function to run the model for each compound
PBK_model_function <- function(pars, ev, inits){
  return(data.frame(PBK_model$solve(pars, ev, inits)))}

#Running the PBK model for each compound, returning a data frame with their results
PBK_result<-parameters %>%
  group_by_all() %>%
  #run the model with the input parameters in the CSV file
  reframe(PBK_model_function(
    pars = c(
      MW = MW,
      Ka = Ka,Fa = Fa,
      Kpad = Kpad, Kpbo = Kpbo, Kpbr = Kpbr,
      Kpgu = Kpgu, Kphe = Kphe, Kpki = Kpki,
      Kpli = Kpli, Kplu = Kplu, Kpmu = Kpmu,
      Kpsk = Kpsk, Kpsp = Kpsp, Kpte = Kpte, Kpre = Kpre,
      SF = SF, fuinc = fuinc, CLint = CLint,
      fup = fup, fuliver = fuliver, BP = BP,
      BW = BW,
      FVad = FVad, FVbo = FVbo, FVbr = FVbr,
      FVgu = FVgu, FVhe = FVhe, FVki = FVki,
      FVli = FVli, FVlu = FVlu, FVmu = FVmu,
      FVsk = FVsk, FVsp = FVsp, FVte = FVte,
      FVve = FVve, FVar = FVar, FVpl = FVpl, FVre = FVre,
      CO = CO,
      FQad = FQad, FQbo = FQbo, FQbr = FQbr,
      FQgu = FQgu, FQhe = FQhe, FQki = FQki,
      FQh = FQh,FQlu = FQlu,FQmu = FQmu,
      FQsk = FQsk,FQsp = FQsp, FQte = FQte, FQre = FQre),
    ev = ev,
    inits =  inits)) #%>%

  #filter(Compound == "Phenethylamine")
  #filter(Compound == "Tyramine")
  #filter(Compound == "P-synephrine")
  #filter(Compound == "Methylsynephrine")
  #filter(Compound == "Isopropyloctopamine")
  #filter(Compound == "P-octopamine")
  #filter(Compound == "Hordenine")
  #filter(Compound == "Higenamine")
  #filter(Compound == "Beta-methylphenethylamine")
  #filter(Compound == "Halostachine")
  #filter(Compound == "Methyltyramine")
  #filter(Compound == "Dimethylphenethylamine")
```

```
# PLOT RESULTS #####
plot_results <- ggplot(PBK_result)+
  geom_line(aes(x=time, y=Cplasmavenous_uM, color = Compound)) +
     labs(y = "Concentration (umol/L)",
       x = "Time (h)")  +
  theme_classic() +
  theme(axis.text = element_text(size = 12, color = "black"))+
  theme(axis.title = element_text(size = 14, color = "black"))

plot_results

#Saving the plot
ggsave(filename = "PBKResultALLCOMPOUNDS_dose-1_BP-simcyp.jpg",width=8, height=4,dpi=300)

# SAVE RESULTS AS CSV file ####
write.csv(PBK_result, "PBKresultALLCOMPOUNDS_dose-1_BP-simcyp.csv")



#####Function to run a sensitivity analysis for a selected compound #####
selectedCompound = "Dimethylphenethylamine"
changeParameter = 1.01 #(increase parameter for the senstivity analysis with 1%)

#load the parameters for the selected compound
unchangedParameters<- parameters %>%
  filter(Compound == selectedCompound)

# starting dataframe with two new columns to add a count for each simulation and to record the
unchanged parameter for later calculations
parametersSensitivity <- unchangedParameters %>%
  mutate(count = 1) %>%
  mutate(unchanged = 0)

#for loop in which each parameter is changed (one-by-one) and a new parameter set is created and
counted
for (i in c(3:54)) {
  add<-c(unchangedParameters[i]*changeParameter,count = i,unchanged =
pull(unchangedParameters[i]),unchangedParameters[-i])
  parametersSensitivity <-rbind(parametersSensitivity,add)
}


#running the PBK model for the sensitivity analysis, returning a data frame with the results (change in
Cmax as a result of the change in input)
PBK_result_sensitivity<-parametersSensitivity %>%
  mutate(Parameter = paste(colnames(unchangedParameters[count]))) %>% #the parameter that has
been changed
  group_by_all() %>%
  reframe(PBK_model_function(
    pars = c(MW = MW,
      Ka = Ka,Fa = Fa,
      Kpad = Kpad, Kpbo = Kpbo, Kpbr = Kpbr,
      Kpgu = Kpgu, Kphe = Kphe, Kpki = Kpki,
      Kpli = Kpli, Kplu = Kplu, Kpmu = Kpmu,
      Kpsk = Kpsk, Kpsp = Kpsp, Kpte = Kpte, Kpre = Kpre,
      SF = SF, fuinc = fuinc, CLint = CLint,
```

```
    fup = fup, fuliver = fuliver, BP = BP,
    BW = BW,
    FVad = FVad, FVbo = FVbo, FVbr = FVbr,
    FVgu = FVgu, FVhe = FVhe, FVki = FVki,
    FVli = FVli, FVlu = FVlu, FVmu = FVmu,
    FVsk = FVsk, FVsp = FVsp, FVte = FVte,
    FVve = FVve, FVar = FVar, FVpl = FVpl, FVre = FVre,
    CO = CO,
    FQad = FQad, FQbo = FQbo, FQbr = FQbr,
    FQgu = FQgu, FQhe = FQhe, FQki = FQki,
    FQh = FQh,FQlu = FQlu,FQmu = FQmu,
    FQsk = FQsk,FQsp = FQsp, FQte = FQte, FQre = FQre),
   ev = ev,
   inits =  inits)) %>%
  group_by(Parameter) %>%
  filter(Cplasmavenous == max(Cplasmavenous)) %>%  ungroup() %>%
  mutate(NSC = ((Cplasmavenous-Cplasmavenous[Parameter == "Compound"])/
                (unchanged*changeParameter-unchanged))*
           (unchanged/Cplasmavenous[Parameter == "Compound"]))%>%
  mutate(Value_changed =  unchanged*changeParameter) %>%
  select(Compound, Parameter, Value = unchanged, Value_changed, Cplasmavenous, NSC)
```

# PLOT SENSITIVITY #####

```
plot_sensitivity<- PBK_result_sensitivity %>%
  filter(Parameter != "Compound") %>%
  filter(abs(NSC)>0.2) %>%
  filter(Parameter != "FQlu") %>% # FQlu is the same as the cardiac output and does not need to be
evaluated separately
  ggplot(., aes(NSC,reorder(Parameter, abs(NSC)))) +
  geom_vline(xintercept = 0) +
  geom_point() +
  #xlim(-1.1, 1.1) +
  labs(y = "Parameter",
       x = "NSC",
       title = selectedCompound)  +
  theme_classic() +
  theme(axis.text = element_text(size = 12, color = "black"))+
  theme(axis.title = element_text(size = 14, color = "black"))
plot_sensitivity
ggsave(filename = paste("SenstivityResult", selectedCompound,".jpg"),width=8, height=4,dpi=300)
```
